# Microglial PGC-1α protects against ischemic brain injury by suppressing neuroinflammation

**DOI:** 10.1186/s13073-021-00863-5

**Published:** 2021-03-26

**Authors:** Bin Han, Wei Jiang, Pan Cui, Kai Zheng, Chun Dang, Junjie Wang, He Li, Lin Chen, Rongxin Zhang, Qing Mei Wang, Zhenyu Ju, Junwei Hao

**Affiliations:** 1 Department of Neurology, Xuanwu Hospital, Capital Medical University, Beijing, 100053 China; 2 Department of Neurology, Tianjin Neurological Institute, Tianjin Medical University General Hospital, Tianjin, 300052 China; 3 Laboratory of Immunology and Inflammation, Department of Immunology and Research Center of Basic Medical Sciences, Key Laboratory of Immune Microenvironments and Diseases of Educational Ministry, Tianjin Medical University, Tianjin, 300070 China; 4 Stroke Biological Recovery Laboratory, Department of Physical Medicine and Rehabilitation, Spaulding Rehabilitation Hospital, the teaching affiliate of Harvard Medical School Charlestown, Boston, MA 02129 USA; 5 Key Laboratory of Regenerative Medicine of Ministry of Education, Institute of Aging and Regenerative Medicine, Jinan University, Guangzhou, 510632 China

**Keywords:** Microglia, Neuroinflammation, PGC-1α, Ischemic stroke

## Abstract

**Background:**

Neuroinflammation and immune responses occurring minutes to hours after stroke are associated with brain injury after acute ischemic stroke (AIS). PPARγ coactivator-1α (PGC-1α), as a master coregulator of gene expression in mitochondrial biogenesis, was found to be transiently upregulated in microglia after AIS. However, the role of microglial PGC-1α in poststroke immune modulation remains unknown.

**Methods:**

PGC-1α expression in microglia from human and mouse brain samples following ischemic stroke was first determined. Subsequently, we employed transgenic mice with microglia-specific overexpression of PGC-1α for middle cerebral artery occlusion (MCAO). The morphology and gene expression profile of microglia with PGC-1α overexpression were evaluated. Downstream inflammatory cytokine production and NLRP3 activation were also determined. ChIP-Seq analysis was performed to detect PGC-1α-binding sites in microglia. Autophagic and mitophagic activity was further monitored by immunofluorescence staining. Unc-51-like autophagy activating kinase 1 (ULK1) expression was evaluated under the PGC-1α interaction with ERRα. Finally, pharmacological inhibition and genomic knockdown of ULK1 were performed to estimate the role of ULK1 in mediating mitophagic activity after ischemic stroke.

**Results:**

PGC-1α expression was shortly increased after ischemic stroke, not only in human brain samples but also in mouse brain samples. Microglia-specific PGC-1α overexpressing mice exhibited significantly decreased neurologic deficits after ischemic injury, with reduced NLRP3 activation and proinflammatory cytokine production. ChIP-Seq analysis and KEGG pathway analysis revealed that mitophagy was significantly enhanced. PGC-1α significantly promoted autophagic flux and induced autolysosome formation. More specifically, the autophagic clearance of mitochondria was enhanced by PGC-1α regulation, indicating the important role of mitophagy. Pharmacological inhibition or knockdown of ULK1 expression impaired autophagic/mitophagic activity, thus abolishing the neuroprotective effects of PGC-1α.

**Conclusions:**

Mechanistically, in AIS, PGC-1α promotes autophagy and mitophagy through ULK1 and reduces NLRP3 activation. Our findings indicate that microglial PGC-1α may be a promising therapeutic target for AIS.

**Supplementary Information:**

The online version contains supplementary material available at 10.1186/s13073-021-00863-5.

## Background

Acute ischemic stroke (AIS) accounts for the majority of catastrophic disabilities and deaths among individuals with cerebrovascular disorders. Unfortunately, effective therapies for AIS remain limited to date [1]. The neuroinflammation and immune responses occurring minutes to hours after stroke were associated with the complex pathology of brain injury after AIS [2, 3]. Many preclinical and clinical studies on stroke have suggested that immune modulation in the central nervous system (CNS) could be a viable alternative treatment strategy for AIS.

Microglia play crucial roles under both physiological and pathological conditions, such as immune surveillance and response to brain injury [4–6]. After stroke, microglia act as the earliest immune responders, substantially contributing to the initiation and propagation of neuroinflammation [7]. Activated microglia promote the infiltration of peripheral leukocytes by releasing proinflammatory cytokines and engulfing adjacent blood vessels [8]. Therefore, selective regulation of microglial activation can strongly suppress brain inflammation.

Peroxisome proliferator-activated receptor-γ coactivator-1α (PGC-1α) is a master coregulator that governs gene expression during mitochondrial biogenesis by interacting with many transcription factors, such as estrogen-related receptor α (ERRα) [9], which also serves as an important coordinator and is involved in the transcriptional regulation of many physiological processes [10, 11]. In addition, PGC-1α has been shown to play a crucial role in various neurological disorders by modulating mitochondrial function and reactive oxygen species (ROS) levels. In multiple sclerosis patients, reduced PGC-1α expression was accompanied by decreased expression of mitochondrial antioxidants and uncoupling proteins (UCPs), thereby leading to neuronal loss and neurodegeneration [12]. In addition, PGC-1α was shown to attenuate MPTP-induced neurodegenerative processes in a Parkinson’s disease (PD) model by upregulating mitochondrial antioxidants, such as GPx1 and SOD2, thereby reducing ROS production [13]. In primary human astrocytes, PGC-1α markedly suppresses oxidative damage and the generation of proinflammatory mediators [14]. However, the role of PGC-1α in microglia in the modulation of poststroke neuroinflammation remains poorly understood.

In this study, we found that PGC-1α expression changed in microglia from stroke patients and a mouse model of AIS. We showed that microglial PGC-1α overexpression could inhibit the neuroinflammatory responses induced by ischemic brain injury. Our results further demonstrated that PGC-1α could promote autophagy and mitophagy through unc-51 like kinase 1 (ULK1), which suppresses hyperactivation of the NLRP3 inflammasome, attenuating neuroinflammation and neurological deficits. Interfering with autophagy and mitophagy using pharmacological inhibition and genetic manipulation abolished the neuroprotective effects of PGC-1α. Thus, our data reveal that targeting microglial PGC-1α may potentially be beneficial for the treatment of AIS.

## Methods

### Human postmortem brain tissue

Postmortem formalin-fixed brain sections were obtained from 4 ischemic stroke patients within 1 day after stroke onset, 4 patients within 3 to 10 days after stroke onset, and 4 age-matched control subjects who died with no documented signs of neurological diseases. These tissues were obtained from the Department of Pathology of Ohio State University (Columbus, OH) and the Brain and Body Donation program of Banner Sun Health Research Institute (Sun City, AZ). Each group included two men and two women. All patients, or their next of kin, in these tissue donation programs gave written informed consent for brain autopsy and the use of brain tissues for research purposes. The stroke lesions were localized at the perfusion territory in the middle cerebral artery. There was no significant difference in age between the controls and the stroke patients (controls: 80.0 ± 5.5 years; stroke patients within 1 day: 86.2 ± 4.7 years; stroke patients within 3 to 10 days: 81.5 ± 4.1 years; *p* > 0.05). The study protocols for brain tissue acquisition were approved by the Institutional Review Boards of Ohio State University and Banner Sun Health Research Institute. The protocols for using these specimens were approved by the Institutional Review Boards of Xuanwu Hospital and Tianjin Medical University General Hospital. The human research in this study also conformed to the principles of the Helsinki Declaration. After the brain specimens were processed, PGC-1α expression in neural cells was detected by immunostaining. Images were captured using a fluorescence microscope. The relative fluorescence intensity was quantified by using ImageJ software.

### Animals

All experimental protocols were performed in compliance with the ARRIVE guidelines and were approved by the Institutional Animal Care and Use Committee of Xuanwu Hospital and Tianjin Medical University General Hospital. All mice used in this study were male and had a C57BL/6 background, were housed under specific pathogen-free conditions with a fixed 12-h light/dark cycle, and were given free access to food and water. Additionally, wild-type mice and homozygous Cas9 knock-in mice were purchased from Vital River Corporation (Beijing, China). PGC-1α^*f/f*^ mice carried a *loxP*-flanked allele of PGC-1α and an enhanced GFP (eGFP) gene and were generously provided by Dr. Zhenyu Ju (Jinan University, Guangzhou, China). For generation of mice with microglia-specific overexpression of PGC-1α (mPGC-1α), PGC-1α^*f/f*^ mice were crossbred with B6.129P2(Cg)-*Cx3cr1*^*tm2.1(cre/ERT2)Litt*^/WganJ mice (Cx3cr1-Cre mice) (Jackson Laboratory stock number 021160), which express tamoxifen (TAM)-inducible Cre recombinase. Littermate *PGC-1α*^*f/f*^ mice were used as control mice. TAM dissolved in corn oil was administered for 5 consecutive days followed by a 30-day interval to allow specific induction before experiments were conducted. To determine the optimal dosage for TAM, we gave the mice 50 to 150 mg/kg either through gavage or intraperitoneal injection. The specificity and efficiency of PGC-1α expression were verified by immunostaining and quantitative real-time polymerase chain reaction (qRT-PCR). For CRISPR-Cas9 editing knockout of PGC-1α, homozygous Cas9 knock-in mice were bred with homozygous Cx3cr1-Cre mice. The offspring of Cx3cr1-Cas9 mice at age 35 days were administered PGC-1α CRISPR AAVs via stereotaxic injection in the cerebral ventricle and, 30 days later, used for molecular and behavioral assays. A total of 357 male mice were included in this study and randomly assigned to the following groups by a lottery drawing: 90 WT mice, 138 PGC-1α^*f/f*^ mice, 109 mPGC-1α mice, and 20 Cx3cr1-Cas9 mice.

### Transient middle cerebral artery occlusion (tMCAO) procedure

Mice at 10–12 weeks underwent a procedure to induce focal cerebral ischemia using silicone rubber-coated 7–0 nylon monofilaments (701956PK5Re; Doccol, Sharon, MA, USA) after TAM induction. Briefly, the mice were anesthetized with chloral hydrate (30 mg/kg), and analgesic gel (lidocaine) was applied evenly on the incision to relieve pain during the experiment. Then, the left common carotid artery, internal carotid artery, and external carotid artery were gently separated from the surrounding tissues. Occlusion was accomplished by slowly inserting monofilaments to block blood flow of the middle cerebral artery. After 60 min of occlusion, the monofilament was withdrawn to allow reperfusion. Throughout the surgery, body temperature was maintained at 37.0 ± 0.5 °C using an electric blanket. For disruption of the expression of ULK1 in the mouse brain, lentiviruses expressing the negative control or ULK1 RNAi were injected into the cortex using a stereotaxic instrument at day 7 before surgery, as our previous study reported [15]. The sham-operated mice were subjected to the same surgical procedures but without the artery occlusion procedures. Regional cerebral blood flow (rCBF) was monitored using laser speckle flowmetry (PeriCam PSI, Stockholm, Sweden) at different time points, including baseline, 30 min after ischemia, and 2 min after reperfusion. The value was expressed as a percentage of the value at baseline. All procedures were performed by the same operator (B.H.) throughout the study to minimize the systemic variation. A series of behavioral tests, including modified neurological severity score (mNSS), corner-turning test, foot-fault test, and rotarod test, were blindly performed to assess neurological deficits, as previously reported [16, 17]. The infarct volume was defined by 2,3,5-tripenyltetrazolium chloride (TTC) staining (Sigma) and quantified by using Image-Pro-Plus 6.0 software (Media Cybernetics, Inc., Rockville, MD, USA). Except for the data analyst, the researchers for neurobehavioral assessment were kept blind to the different genotypes of mice, outcome measurements, and trial results.

### Immunofluorescence staining

Brain sections were permeabilized with 0.3% Triton X-100 for 10 min and then washed. Nonspecific staining was blocked with 3% BSA for 1 h. The sections were incubated with primary antibodies overnight at 4 °C. Primary antibodies included rabbit anti-PGC-1α (1:300, Abcam), goat anti-Iba-1 (1:800, Abcam), mouse anti-eGFP (1:250, Abcam), rabbit anti-Iba-1 (1:500, Wako), rabbit anti-GFAP (1:1000, Abcam), rabbit anti-NeuN (1:500, Abcam), rabbit anti-NLRP3 (1:50, Abcam), rabbit anti-ASC (1:50, Santa Cruz), goat anti-GFAP (1:1000, Abcam), and mouse anti-NeuN (1:1000, Abcam). After incubation with primary antibodies, the sections were washed with PBS (5 × 5 min) and then incubated with appropriate secondary antibodies at room temperature for 1 h. For the analysis of neuronal apoptosis, brain sections were incubated with TUNEL reagents following the manufacturer’s guidelines.

### Analysis of microglial morphology

After TAM induction, brains were removed from the PGC-1α^*f/f*^ and mPGC-1α mice. Subsequently, 50-μm coronal sections were cut. After permeabilization and blocking, the sections were incubated with rabbit anti-Iba-1 primary antibody (1:500, Wako) for 48 h at 4 °C. The sections were washed with PBS and then incubated with Alexa Fluor 488-conjugated donkey anti-rabbit secondary antibody (1:500; A-21206; Thermo Fisher Scientific) for 24 h at 4 °C in the dark. For image analysis, microglia in the cortex were scanned step by step at a thickness of 1 μm in the *z* direction using a confocal microscope (Olympus, Heidelberg, Germany). Three-dimensional images were reconstructed using Imaris software (Bitplane), and morphological data were then semiautomatically calculated using this software.

### Flow cytometry

Fresh single-cell suspensions were prepared for fluorescence-activated cell sorting (FACS) analysis. The mononuclear cells in the brains were separated with 30% Percoll. After cells were blocked with 1% BSA, they were incubated with fluorochrome-conjugated antibodies against IL-6 or TNF-α in the dark. Anti-mouse IL-1β (Cell Signaling Technology) and anti-mouse NLRP3 (Abcam)-labeled cells were detected with the corresponding fluorochrome-conjugated secondary antibodies. The antibodies were tagged with PE, APC, or PerCP. Flow cytometry was performed using a FACSAria Cell Sorter (BD Biosciences, San Jose, CA, USA), and then, the acquired data were analyzed with FlowJo® vers. 7.6 software (Ashland, OR, USA).

### Microglial isolation and sorting

Primary microglia were isolated using a MojoSort™ mouse P2RY12 selection kit (Biolegend, San Diego, CA, USA) according to the manufacturer’s protocols. Briefly, brains were minced and filtered with a 70-μm cell strainer on ice and then centrifuged at 2000 rpm for 5 min. Myelin and cell debris were removed by using 30% Percoll gradient centrifugation. The cell concentration was adjusted to 1 × 10^7^ cells/ml. Subsequently, the cells were incubated with biotin-conjugated P2RY12 antibody (10 μl/10^6^ cells) on ice for 15 min and then incubated with streptavidin-conjugated nanobeads on ice for 15 min. After washing, the cell suspension was placed into a MojoSort™ magnetic separation system on ice for 5 min. Finally, the positive P2RY12-labeled cells on the tube wall were identified as microglia.

### Microarray analysis

Total RNA was extracted from primary microglia and BV2 cells and purified by using a NucleoSpin® RNA clean-up kit (Machery- Nagel, Germany). The concentration and purity of the RNA samples were measured with a Qubit fluorometer (Invitrogen), and the integrity was determined by agarose gel electrophoresis and a BioAnalyzer 2100 system (Agilent RNA 6000 Nano kit). After cRNA amplification, cDNA was synthesized and labeled with Cy5-dCTP (GE Healthcare). The labeled cDNA was hybridized to Mouse Gene Expression 8 × 60 k v2 microarrays (Agilent Technologies). The raw data were imported into GeneSpring software V13 (Agilent Technologies), and then, normalization for quality control was carried out. Cluster analysis was performed using Cluster 3.0 software with log2 transformation and hierarchical clustering by average linkage. The visual adjustment and graphic output of cluster diagrams were achieved using Java TreeView microarray visualization software (Stanford University School of Medicine, Stanford, CA, USA). The microarray data were deposited in the NCBI Gene Expression Omnibus (GEO) (GSE124874, GSE152769, and GSE152871) [18–20].

### Mouse inflammatory cytokine array

For determination of whether PGC-1α could alter the cytokine profiles of microglia after ischemic stroke, 40 inflammatory cytokines were quantified by using a RayBio protein array (AAM-INF-G1, RayBiotech, Norcross, GA, USA). Microglia were isolated from the PGC-1α^*f/f*^ and mPGC-1α mice at 24 h after stroke. Equal amounts of proteins extracted from microglia were added to the RayBio protein array following the manufacturer’s guidelines. The relative expression levels of various cytokines were quantified by densitometry, and the data were processed using HemI 1.0 software.

### Chromatin immunoprecipitation-sequencing (ChIP-Seq) assay

For identification of the targets of PGC-1α in microglia, microglia were isolated from the mPGC-1α mice at 24 h after tMCAO. After crosslinking with formaldehyde and DSG, cell nuclei were extracted. Then, chromatin was sheared by sonication and immunoprecipitated using a ChIP-grade anti-GFP antibody (Abcam, ab290). According to the TF Antibody Characterization Standards (May 2016), ab290 was characterized and met the standards set by the ENCODE Consortium. The separated DNA sample was used to perform ChIP-Seq assays. Briefly, DNA end-fragment end repair, addition of Klenow exo- with dATP, and adapter ligation were performed by using a Paired-End DNA Sample Prep kit (Illumina). After PCR amplification, the DNA was sequenced on an Illumina HiSeq 4000 system according to the HiSeq 3000/4000 SBS kit (300 cycles) protocol. The read depth was > 20 million per sample, which met the target-specific standard of 20 million usable fragments per replicate. Raw reads were aligned to the mouse genome (UCSC MM10) using BOWTIE software (V2.1.0) with default parameters. The mapped reads were used to identify peak regions using MACS V1.4.2 software (Model-based Analysis of ChIP-Seq). Finally, we analyzed the distribution of peak regions in the genome sequence and peak enrichment in repeat classification. Two samples were used for the sequence analysis. For the ChIP-Seq data analysis, the raw data were trimmed by Trimmomatic (version 0.36) software and then aligned with the mm10 genome by BOWTIE2 (version 2.3.4.1). Duplications were then removed from the alignment results by Picard (version 2.23.8). After alignment of the files basic statistics, the peak calling was processed by MACS2 (version 2.2.7.1) software against the input sample, with a *p* value < 0.001 [21]. The ENCODE blacklist of the mm10 genome (downloaded from https://www.encodeproject.org/annotations/ENCSR636HFF/) was blocked by BEDOPS software (version 2.4.39) with the -n1 parameter [22, 23]. Thus, peaks reappearing in two ChIP samples were obtained by idr (version 2.0.4.2). Sites yielding peaks across both replicates were used for the subsequent analysis. Peaks were annotated by HOMER (v 4.11.1) annotatePeaks.pl script. HOMER motif analysis was conducted by using HOMER’s (v 4.11.1) findMotifsGenome.pl and the fragment size was set as “given” and length was set as default. Then, the de novo motifs were compared to the JASPAR (2020) vertebrate nonredundant database by HOMER compareMotifs.pl [24]. To verify the ChIP-Seq data, we used the DNA obtained from ChIP for ChIP-qPCR. The primers are shown in Additional file 1: Table S1. The ChIP-Seq data were deposited in the NCBI GEO database (Accession # GSE165564) [25].

### Cell culture, lentiviral transfection, and treatment

BV2, a microglial cell line, was cultured with DMEM/F-12 (1:1, Thermo Fisher Scientific) containing 10% fetal bovine serum (FBS, Life Technologies, Vienna, Austria) and 1% penicillin/streptomycin (Life Technologies) in 5% CO_2_ at 37 °C. For overexpression of PGC-1α in BV2 cells, the target gene fragment was amplified with the following primers: 5′- GAGGATCCCCGGGTACCGGTCGCCACCATGGCTTGGGACATGTGCAG − 3′ (forward) and 5′- TCCTTGTAGTCCATACCCCTGCGCAAGCTTCTCTGAGCTTC − 3′ (reverse). Then the fragment was cloned into the lentiviral vector Ubi-MCS-3FLAG-SV40-EGFP-IRES-puromycin (Genechem Co., Ltd., Shanghai, China). After successful construction, lentiviruses containing the target fragment or negative control were transfected into BV2 cells. PGC-1α-overexpressing BV2 cells were selected with puromycin (2 μg/ml; Santa Cruz) after 72 h of transfection. To determine the influence of PGC-1α on inflammatory cytokines, we stimulated BV2 cells with 500 ng/ml LPS (Sigma Aldrich, St. Louis, MO, USA) for 6 h. NLRP3 was activated by LPS induction for 6 h followed by 2 mM ATP stimulation (InvivoGen, San Diego, CA, USA) for 3 h.

### Luciferase assay

BV2 cells were seeded in 24-well plates and cotransfected with ULK1 luc (1 μg), pcDNA3.1-PGC-1α plasmid (1 μg), and pcDNA3.1-ERRα plasmid (1 μg) using Lipofectamine 2000 and then treated with XCT790 (10 μM). The pGL3 plasmid (1 μg)-transfected group was used as a control. After 48 h of transfection, cells were harvested, and then, luciferase activities were determined using a Dual-Luciferase Reporter Assay kit (Promega, Germany) according to the manufacturer’s instructions.

### Oxygen and glucose deprivation (OGD) treatment

OGD was used to mimic ischemic conditions for microglia in vitro. Briefly, lentiviruses carrying the control, PGC-1α, PGC-1α-RNAi, or ULK1-RNAi were transfected into BV2 cells. These vectors were labeled with puromycin, but without a GFP tag. Then, the BV2 cells were incubated with puromycin to remove the untransfected cells. For evaluation of autophagy, Ad-mRFP-GFP-LC3 was transfected into BV2 cells. For assessment of mitophagy, BV2 cells were transfected with Ad-GFP-LC3 or COX8-EGFP-mCherry lentivirus. After successful transfection, the BV2 cells were incubated in a self-contained and sealed hypoxia incubator chamber (Cat. 27310; StemCell Technologies Inc) (5% CO_2_ and 95% N_2_) for 2 h in DMEM without serum and glucose. Then, the BV2 cells were cultured in normal medium at 37 °C for 24 h, followed by examination of fluorescence under a confocal microscope, and the number of puncta was calculated using ImageJ software.

### Transmission electron microscopy (TEM)

TEM was used to assess mitophagy. Briefly, BV2 cells were cultured and treated as described above. After OGD treatment, BV2 cells were fixed with 2.5% glutaraldehyde for 2 h at 37 °C and 1% osmium tetraoxide for 1 h. Thereafter, BV2 cells were subjected to gradient dehydration in ethanol and embedded in Spurr resin. Next, cells were cut into 70-nm ultrathin sections and collected on nickel grids, followed by staining with uranyl acetate and lead citrate. Finally, cells were photographed using a transmission electron microscope (JEM-1230, JEOL, Japan), and the images were analyzed with ImageJ software.

### Detection of ROS generation in vivo and in vitro

Dihydroethidium (DHE) (Sigma), a superoxide indicator, was used to detect in situ ROS production in the ischemic penumbra. DHE could react with superoxides and was then oxidized to the red fluorescent molecule ethidium. At 24 h after tMCAO, mice were injected with DHE (100 μl; 4 mg/ml) for 3 consecutive hours. Next, the brain sections were examined under a fluorescence microscope. The relative DHE fluorescence intensity was quantified using ImageJ software.

Intracellular ROS levels in BV2 cells were measured using a Reactive Oxygen Species Assay kit (Beyotime Institute of Biotechnology, Nanjing, China) according to the manufacturer’s guidelines. In brief, after OGD, we used a fluorimetric probe, 2′,7′-dichlorodihydrofluorescein-diacetate (DCFH-DA), to detect ROS. BV2 cells were incubated with DCFH-DA for 20 min in the dark and then oxidized to dichlorofluorescein. The mean fluorescence intensity was quantified by a fluorospectrophotometer.

### Determination of SOD activity and MDA levels

Brains were collected from the PGC-1α^*f/f*^ and mPGC-1α mice after tMCAO, and then were homogenized. The total protein concentration was measured by using a BCA protein assay kit. Subsequently, the SOD activity and MDA content were measured by using SOD and MDA assay kits (Nanjing Jiancheng Bioengineering Institute, Nanjing, China), respectively, according to the manufacturers’ instructions. All data were calculated and normalized to the total protein concentration in each sample.

### Seahorse assay

The oxygen consumption rate (OCR) of BV2 cells was determined by a Seahorse Bioscience XF^e^96 Extracellular Flux Analyzer using a XF Cell Mito Stress Test Kit (Seahorse Bioscience, USA) according to the manufacturer’s instructions, as we described previously [26]. After OGD treatment, BV2 cells were incubated in CO_2_-free incubator. Then the OCR and key parameters of metabolic function were recorded with successive stimulation with oligomycin (1 μM), FCCP (0.5 μM), and rotenone/antimycin A (1 μM). Finally, basal respiration, ATP production, maximal respiration, spare capacity, and proton leak were calculated.

### Isolation of primary neurons, microglia and astrocytes

Primary microglia, astrocytes, and neurons were isolated from 1-day-old neonatal PGC-1α^*f/f*^ and mPGC-1α mice. Briefly, the brains were gently removed, and then, the meninges, cerebellum, and brainstem were completely dissected away on ice. The remaining brain tissues were placed into Hank’s buffer and digested with trypsin and DNase. For the isolation of primary microglia and astrocytes, the cell suspension was filtered with a 40-μm cell strainer and then centrifuged. The pelleted cells were subsequently suspended in DMEM/F-12 and seeded into flasks precoated with poly-l-lysine. The medium was changed every 2 days, and the cells were cultured at 37 °C for 7 to 10 days. The microglia and astrocytes were separated from the mixed glial cell cultures by shaking the flasks at 37 °C for 4 h. Finally, the microglia were left floating in suspension while the astrocytes remained adherent to the flask. For the isolation of primary neurons, the resuspended cells were seeded in poly-l-lysine-pretreated 100 dishes containing high-glucose DMEM. After 4 h, the medium was changed to neurobasal medium (Thermo Fisher Scientific) containing 2% B27, 1% glutamine, 1% HEPES, and 1% penicillin/streptomycin. Half of the neurobasal medium was changed every 3 days, and the neurons were cultured for 7 to 10 days. The isolated microglia, astrocytes, and neurons were all treated with TAM (0.4 mg/ml) for 3 days. Then, these cells were collected to extract total RNA for detection of PGC-1α mRNA expression.

### Enzyme-linked immunosorbent assay (ELISA)

After LPS and ATP stimulation, BV2 cell supernatants were collected. Inflammatory cytokines, including IL-1β, IL-6, and TNF-α, were then measured by ELISA (Neobioscience, Shenzhen, China) according to the manufacturer’s instructions. All samples and standards were determined in duplicate.

### qRT-PCR

Total RNA was extracted from fresh brain, spleen, liver, kidney, heart, lung, or cultured cells by using TRIzol reagent (Invitrogen, Carlsbad, CA, USA) according to the manufacturer’s guidelines. Then, cDNA was synthesized using TransScript First-Strand cDNA Synthesis Super Mix (TransGen Biotech, China). qPCR reactions were performed in triplicate with FastStart Universal SYBR Green Master Mix (Roche, Germany) on a CFX ConnectTM Real-Time PCR Detection System (Bio-Rad, USA). Relative gene expression was normalized to the housekeeping gene GAPDH and calculated using the 2^−ΔΔCt^ method. The primers used for amplification are shown in Additional file 1: Table S2.

### Western blots

Brain tissues and cultured cells were harvested and then lysed with RIPA buffer (Solarbio, China) containing cOmplete™ protease inhibitor cocktail (Roche). Proteins were electrophoretically separated on 10% SDS-PAGE gels and then transferred onto PVDF membranes (Millipore, USA). After blocking with 5% nonfat milk, the membranes were incubated with primary antibodies overnight at 4 °C. The primary antibodies included anti-PGC-1α (1:1000, Thermo Fisher), anti-LC3 (1:1000, Cell Signaling Technology), anti-ERRα (1:1000, Cell Signaling Technology), anti-ULK1 (1:1000, Cell Signaling Technology), anti-sequestosome1 (SQSTM1) (1:1000, Cell Signaling Technology), anti-TOMM20 (1:1000, Cell Signaling Technology), anti-COX IV (1:1000, Abcam), anti-IMMT (1:1000, Abcam), anti-NLRP3 (1:1000, AdipoGen), anti-ASC (1:200, Santa Cruz), and anti-IL-1β (1:800, AdipoGen). After washing, the specific blots were incubated with the species-appropriate secondary antibodies for 1 h at room temperature. Finally, the protein bands were viewed with a Gel Doc 2000 imaging system (Bio-Rad, USA) and then analyzed with the ImageJ software. In the quantitative analysis of Western blots, all the bands detected were within the linear range of detection.

### Statistical analysis

Data were analyzed using GraphPad Prism (GraphPad Software, Version 5.0, La Jolla, CA, USA) and shown as the median and interquartile range. The Mann-Whitney *U* test was used to compare the difference between two groups. One-way ANOVA (Kruskal-Wallis test) followed by an appropriate post hoc test was used to analyze the differences among multiple groups. The percentage of survival was evaluated using a log-rank (Mantel-Cox) test. *P* < 0.05 was considered statistically significant.

## Results

### PGC-1α expression fluctuates in a time-dependent manner after ischemic stroke

**Fig. 1:**
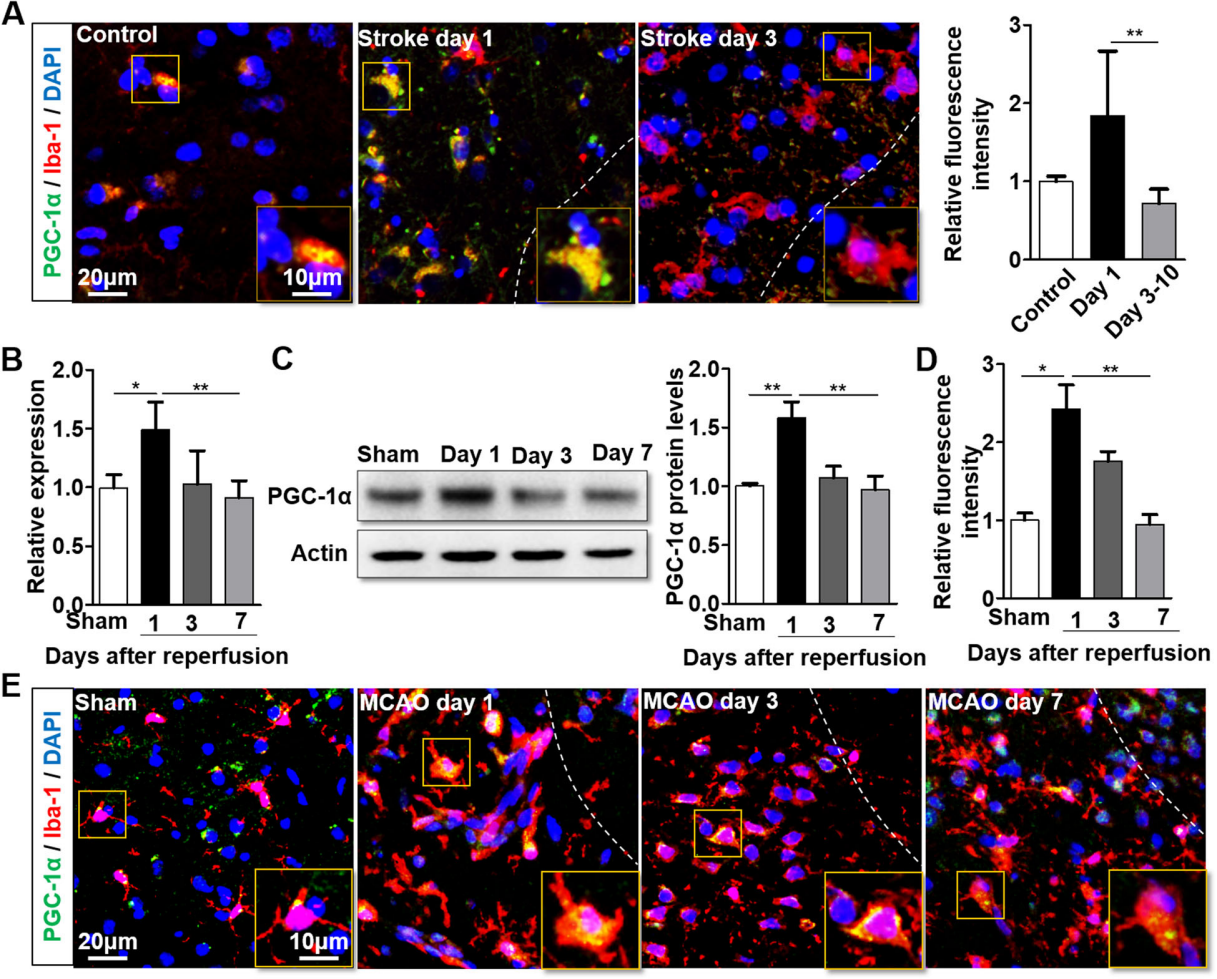
PGC-1α expression fluctuates in a time-dependent manner after ischemic stroke **a** Representative images showing the PGC-1α levels in microglia from ischemic stroke patients (left panel). Quantification of PGC-1α expression by ImageJ software (right panel). *n* = 4 per group. Determination of PGC-1α mRNA (**b**) and protein expression (**c**) in isolated microglia from mice after tMCAO. **d**, **e** Representative images and quantification of relative fluorescence intensity showing microglial PGC-1α expression in ischemic stroke mice. **a, e** The dashed line divides the infarction core and penumbra regions. *p < 0.05, **p < 0.01; **b–e** *n* = 6 per group

To date, the role of PGC-1α in microglia after AIS is still unclear. To address this challenge, we first evaluated the levels of PGC-1α in postmortem brain samples from ischemic stroke patients. We found that PGC-1α expression was altered at different time points after ischemic stroke. Analysis of the relative fluorescence intensity showed that the PGC-1α expression in microglia increased at day 1 after stroke onset compared to that of the controls with non-neurological disease, but decreased in the patients who died on days 3–10 after stroke onset (Fig. 1a). Correspondingly, the mRNA and protein levels of PGC-1α in the microglia from mice also peaked at day 1 but gradually declined at days 3–7 after tMCAO (Fig. 1b, c). In addition, immunofluorescence staining further verified these results, demonstrating that PGC-1α expression in microglia was altered in a time-dependent manner in the tMCAO model (Fig. 1d, e). Furthermore, the levels of PGC-1α in the neurons and astrocytes of humans and mice with AIS were determined, and we found that PGC-1α expression was downregulated in both neurons and astrocytes at day 1 after AIS (Additional file 2: Fig. S1). Taken together, our findings indicate that PGC-1α in microglia may participate in the pathophysiology of AIS.

### Inducible microglia-specific overexpression of PGC-1α via the Cre-loxP system

To further determine the role of PGC-1α in microglia, we conditionally overexpressed PGC-1α in microglia through genetic manipulation. To achieve this, we crossed PGC-1α^*f/f*^ mice, which harbor the *Pgc-1α* gene linked to a fluorescent tag (IRES-eGFP), and Cx3cr1-Cre/ER mice to produce a hybrid capable of specifically overexpressing PGC-1ɑ in microglia in a TAM-induced manner. The gene targeting strategy for generating PGC-1α^*f/f*^ Cx3cr1-Cre/ER (mPGC-1α) mice is shown in Additional file 2: Fig. S2a. The hybrid mice were used for tMCAO experiments 30 days after TAM induction, which ensures the specific overexpression of PGC-1α in microglia rather than macrophages [27]. To optimize the TAM dosage regimen and choose the most effective drug delivery route (gavage vs. intraperitoneal [i.p.]), we first evaluated the safe dosage of each means of delivery by monitoring the weight of the mice from different groups (Additional file 2: Fig. S2b). Further immunostaining showed that PGC-1α expression increased with increasing TAM dosage and was at a relatively steady state when given with TAM (75 mg/kg, i.p.) for 5 consecutive days (Additional file 2: Fig. S2c). Thus, 75 mg/kg TAM was selected for the subsequent experiments. Moreover, PGC-1α expression in the mPGC-1α mice was approximately twofold higher than that in the PGC-1α^*f/f*^ mice (Additional file 2: Fig. S2d).

Next, immunofluorescence staining of eGFP with Iba-1, GFAP, and NeuN demonstrated that PGC-1α was specifically overexpressed in microglia from the mPGC-1α mice (Additional file 2: Fig. S3a). qRT-PCR analysis also confirmed that PGC-1α mRNA expression increased approximately 2-fold in microglia (Additional file 2: Fig. S3b). Moreover, there were no significant differences in PGC-1α mRNA expression in the spleen, liver, kidney, heart, and lung tissues from the mPGC-1α mice compared to the PGC-1α^*f/f*^ controls (Additional file 2: Fig. S3c). Together, these results indicate that microglia from the mPGC-1α mice specifically overexpress PGC-1α. This conclusion bolstered our confidence to determine the role of microglial PGC-1α in the pathophysiology after AIS.

### Microglial PGC-1α protects against ischemia-induced brain damage in mice

**Fig. 2:**
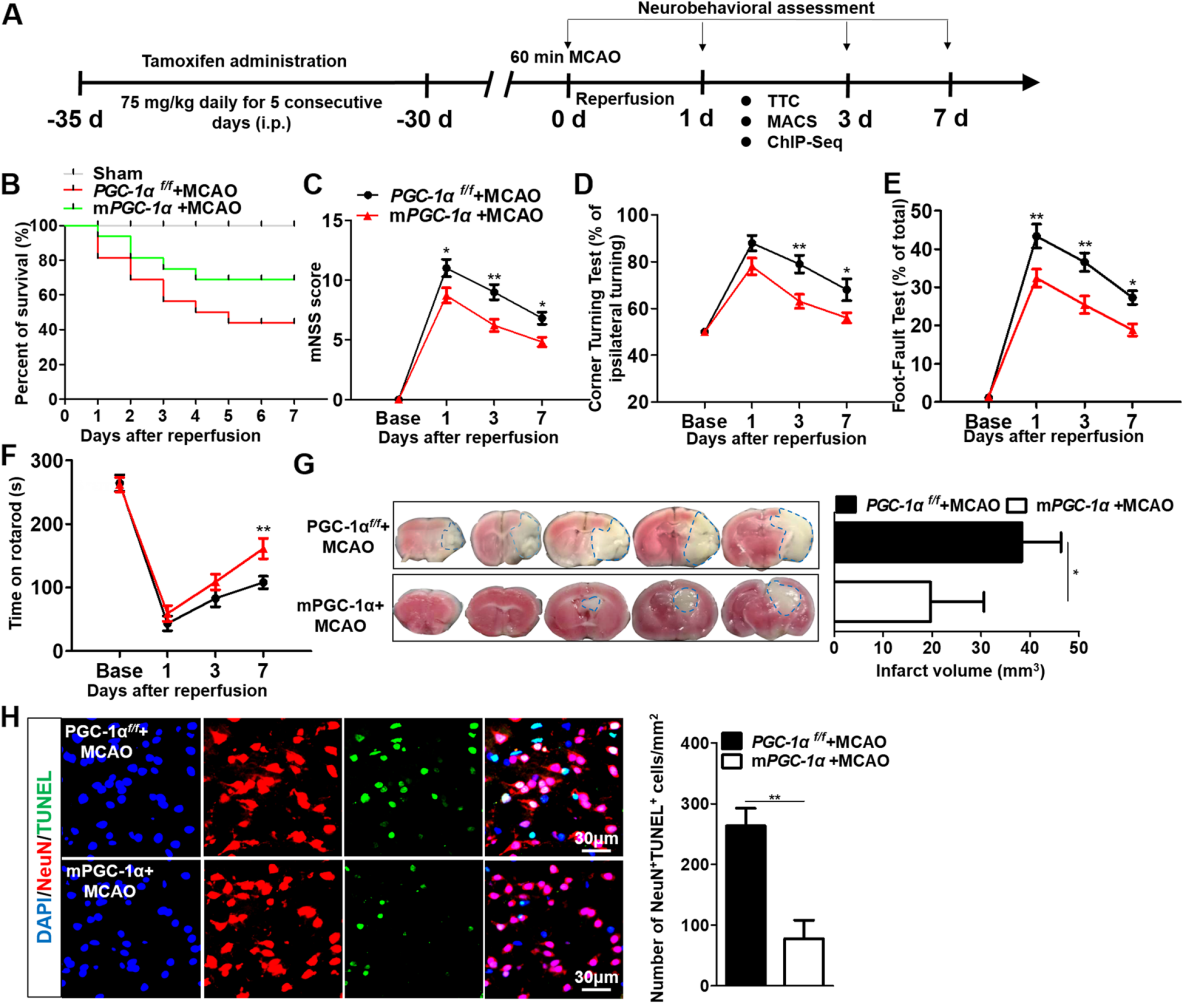
Microglial PGC-1α protects against ischemia-induced brain damage in mice. **a** The schematic of experimental protocols. **b** The percent survival of PGC-1α^*f/f*^ and mPGC-1α mice after tMCAO. *n* = 16 per group. The mNSS score (**c**), corner-turning test (**d**), foot-fault test (**e**), and time on rotarod (**f**) were used to evaluate the neurological functions of motor, sensory, and balance between the PGC-1α^*f/f*^ and mPGC-1α mice. *n* = 10 per group. **g** Infarct size was defined by TTC staining (left panel) and quantified using Image-Pro-Plus 6.0 software (right panel). *n* = 7 per group. **h** Analysis of neuronal apoptosis in the PGC-1α^*f/f*^ and mPGC-1α mice using TUNEL staining (left panel). Quantification of TUNEL/NeuN double-positive cells using ImageJ software (right panel). *p < 0.05, **p < 0.01; *n* = 6 per group

For determination of the impact of microglial PGC-1α overexpression on ischemic brain injury, the mPGC-1α transgenic mice and the littermate controls of PGC-1α^*f/f*^ mice underwent the tMCAO procedure after TAM treatment. A variety of neurobehavioral tests were subsequently performed at days 1, 3, and 7 after tMCAO (Fig. 2a). To exclude the variations in vascular obstruction between these two groups of mice during tMCAO operation, we measured rCBF using laser speckle flowmetry and verified that no significant differences were observed in rCBF at baseline, during ischemia, or reperfusion (Additional file 2: Fig. S4). Interestingly, the mPGC-1α mice had a significantly lower mortality rate than the littermate controls after tMCAO (Fig. 2b). The neurological scores were all significantly decreased in the mPGC-1α mice compared to the PGC-1α^*f/f*^ mice (Fig. 2c–e). Moreover, the accelerated rotarod test revealed that the mPGC-1α mice had better motor and sensory functions and better balance skills than the PGC-1α^*f/f*^ mice (Fig. 2f). TTC staining of brain sections revealed that infarct volume decreased in the mPGC-1α mice compared to the PGC-1α^*f/f*^ mice (Fig. 2g). We assessed mice with microglia-specific PGC-1α knockout by CRISPR-Cas9 editing after AIS. The neurological behavior evaluation performed 3 days after stroke on these mice revealed that microglial PGC-1α knockout exacerbated the severity of stroke in the acute stage (Additional file 2: Fig. S5). Furthermore, we found that the mPGC-1α mice exhibited better clinical scores at day 14 after tMCAO than the PGC-1α^*f/f*^ mice (Additional file 2: Fig. S6), which suggested the potential neuroprotective impact of microglial PGC-1α on neural recovery in ischemic brain injury. Moreover, the mPGC-1α mice exhibited fewer neuronal apoptotic events than the PGC-1α^*f/f*^ mice (Fig. 2h). To uncover the effect of PGC-1α on the interaction between microglia and neurons, we conducted a Transwell experiment. Primary microglia and neurons were isolated from 1-day-old neonatal PGC-1α^*f/f*^ and mPGC-1α mice. After inflammatory stimulation, we found that the neurons cocultured with microglia from the mPGC-1α mice showed substantially reduced neuronal apoptosis under inflammatory conditions in Transwell chambers, and these neurons had normal-looking axons and dendrites, suggesting that PGC-1α overexpression in microglia affects the interaction with neurons and protects neurons against inflammatory insults (Additional file 2: Fig. S7). These data collectively suggest that microglia-specific PGC-1α overexpression plays a key role in limiting ischemia-induced brain damage.

### PGC-1α enhances microglial ramification and alters gene expression profiles

**Fig. 3:**
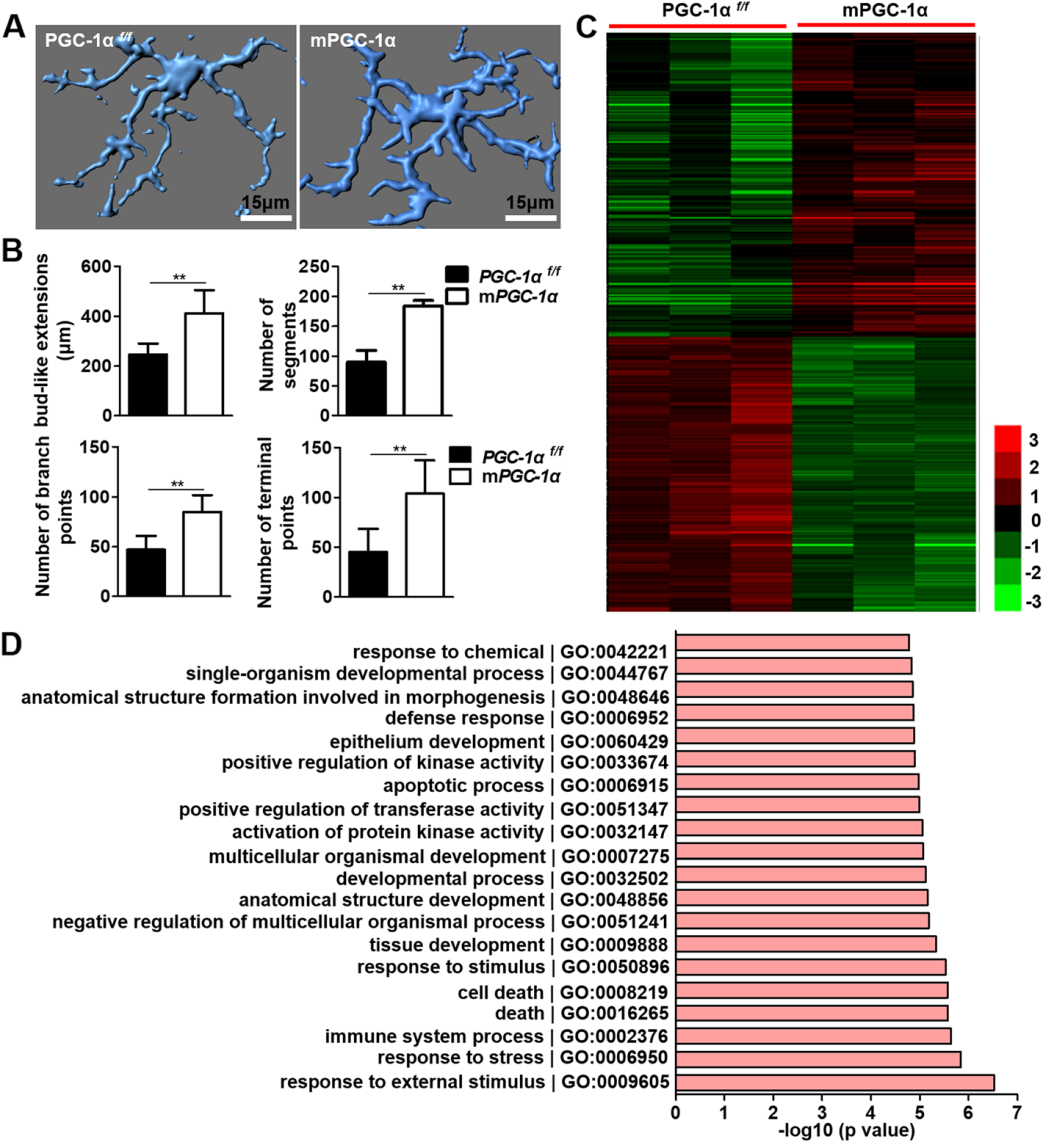
PGC-1α enhances microglial ramification and alters gene expression profiles. **a** Representative images of Imaris-based three-dimensional reconstruction of Iba-1^+^ microglia from PGC-1α^*f/f*^ and mPGC-1α mice. **b** Morphological features, including dendrite length, number of segments, branch points, and terminal points were quantified using Imaris. **c** A total of 260 upregulated and 232 downregulated genes were identified using microarray analysis of microglia. **d** GO analysis showed the top 20 enriched biological processes. **p < 0.01; **a, b** *n* = 5 per group, **c, d** *n* = 3 per group

To investigate the underlying mechanism of PGC-1α-mediated neuroprotection after stroke, we first determined whether PGC-1α overexpression could innately alter microglial function. We assessed the morphology of Iba-1^+^ microglia from the cortex of the PGC-1α^*f/f*^ and mPGC-1α mice using confocal microscopy, and then, the images were reconstructed in three dimensions by Imaris software. Interestingly, the morphology of microglial ramifications was significantly different between these two groups of mice in the resting state. The processes of microglia from the mPGC-1α mice were more ramified than those from the PGC-1α^*f/f*^ mice (Fig. 3a). Moreover, the altered morphology was verified by increased bud-like extensions, number of segments, branch points, and terminal points in the mPGC-1α mice (Fig. 3b). Thus, PGC-1α was proven to efficiently increase the ramified processes of microglia, which potentially enhances their surveillance function and makes them more rapidly sense and respond to ischemic insults [4]. We further assessed the morphology of Iba-1^+^ microglia in the penumbra of the PGC-1α^*f/f*^ and mPGC-1α mice after AIS. As shown in Additional file 2: Fig. S8, we found that there were more branches in microglia from the mPGC-1α mice than in those from the PGC-1α^*f/f*^ mice. However, the soma of microglia from the PGC-1α^*f/f*^ mice was larger than that from the mPGC-1α mice. These data indicated that PGC-1α decreases the activation degree of microglia under ischemic stimulus. To uncover the functional mechanism of PGC-1α, we also performed microarray analysis using sorted microglia from the PGC-1α^*f/f*^ and mPGC-1α mice. Specifically, a total of 260 genes were upregulated and 232 genes were downregulated in microglia of the mPGC-1α mice compared to the PGC-1α^*f/f*^ mice (Fig. 3c), indicating that PGC-1α alters the gene expression profiles of microglia. Subsequently, Gene Ontology (GO) analysis was conducted on these differentially expressed genes. Several biological processes, such as response to external stimulus, response to stress, immune system process, apoptotic process, defense response, and anatomical structure formation involved in morphogenesis, were identified (Fig. 3d). We also performed microarray analysis using sorted microglia from the PGC-1α^*f/f*^ and mPGC-1α mice after AIS. The data revealed that PGC-1α altered metabolism- and morphogenesis-related genes after AIS (Additional file 2: Fig. S9). Taken together, these data suggest that PGC-1α inherently regulates microglial function and allows cells to respond more efficiently after ischemic stroke.

### PGC-1α attenuates neuroinflammation by suppressing NLRP3 hyperactivation

**Fig. 4:**
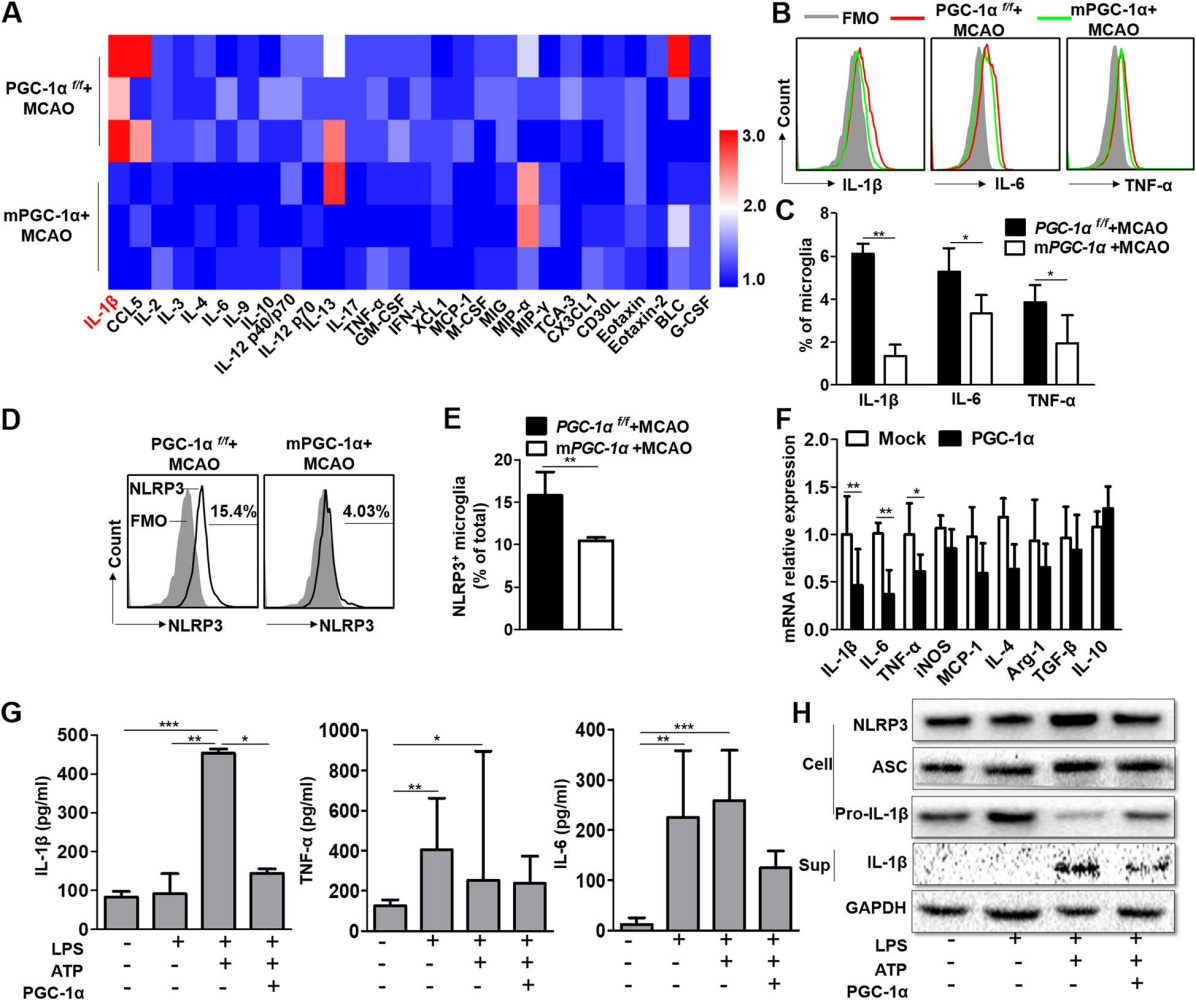
PGC-1α attenuates neuroinflammation by suppressing NLRP3 hyperactivation. **a** Expression of various cytokines/chemokines in microglia by protein array analysis. **b** FACS analysis of IL-1β, IL-6, and TNF-α levels in microglia from the two types of mice after tMCAO. **c** Quantification of IL-1β, IL-6, and TNF-α levels based on FACS analysis. **d** FACS analysis of NLRP3 expression in microglia from the PGC-1α^*f/f*^ and mPGC-1α mice at 24 h after tMCAO. **e** Quantification of NLRP3 expression based on FACS. **f** The mRNA expression of inflammatory cytokines was detected in BV2 cells after LPS stimulation for 6 h. **g** Quantification of IL-1β, IL-6, and TNF-α levels by ELISA. **h** Western blot analysis of NLRP3, ASC, and pro-IL-1β in cellular lysates and IL-1β in supernatant. *p < 0.05, **p < 0.01, ***p < 0.001; **a** *n* = 3 per group, **b–e** *n* = 6 per group, **f–h** *n* = 5–10 per group

To determine the effects of PGC-1α on inflammatory reactions in the CNS, we first employed protein array analysis to quantify 40 cytokines/chemokines in microglia. Many proinflammatory cytokines, such as CCL5, IL-6, IL-17, and TNF-α, were decreased in microglia from the mPGC-1α mice compared to those from the PGC-1α^*f/f*^ mice. In addition, IL-1β was obviously decreased in the mPGC-1α mice (Fig. 4a). To confirm these results, we conducted FACS analysis to measure the levels of IL-1β, IL-6, and TNF-α. As expected, the levels of IL-1β, IL-6, and TNF-α all decreased in microglia from the mPGC-1α mice (Fig. 4b, c). Previous studies have suggested that the NLRP3 inflammasome plays a crucial role in amplifying the inflammatory response [28]. FACS analysis indicated that NLRP3 activation was also inhibited in microglia from the mPGC-1α mice (Fig. 4d, e). To confirm the results obtained from in vivo experiments, we examined the levels of IL-1β, IL-6, and TNF-α in the PGC-1α-overexpressing BV2 cells and found they were decreased (Fig. 4f, g), along with reduced NLRP3, ASC, and pro-IL-1β expression (Fig. 4h). In addition, the gene expression profiles were assessed in BV2 cells and PGC-1α-overexpressing BV2 cells after LPS stimulation, and the results indicated that PGC-1α altered the metabolism and inflammation-related genes in BV2 cells (Additional file 2: Fig. S10). Furthermore, the effects of PGC-1α on inflammatory cytokine production and inflammasome activation in BV2 cells were evaluated both in the absence and presence of LPS insult. We found that there was no significant difference in inflammatory cytokine production and inflammasome activation between BV2 cells and PGC-1α-overexpressing BV2 cells without LPS stimulation. However, the inflammatory responses were reduced in the PGC-1α-overexpressing BV2 cells compared with the BV2 cells after LPS stimulation (Additional file 2: Fig. S11). These data indicated that PGC-1α could inhibit NLRP3 activation and subsequently reduce the production of proinflammatory cytokines.

### Identification of genome-wide transcriptional targets of PGC-1α

**Fig. 5:**
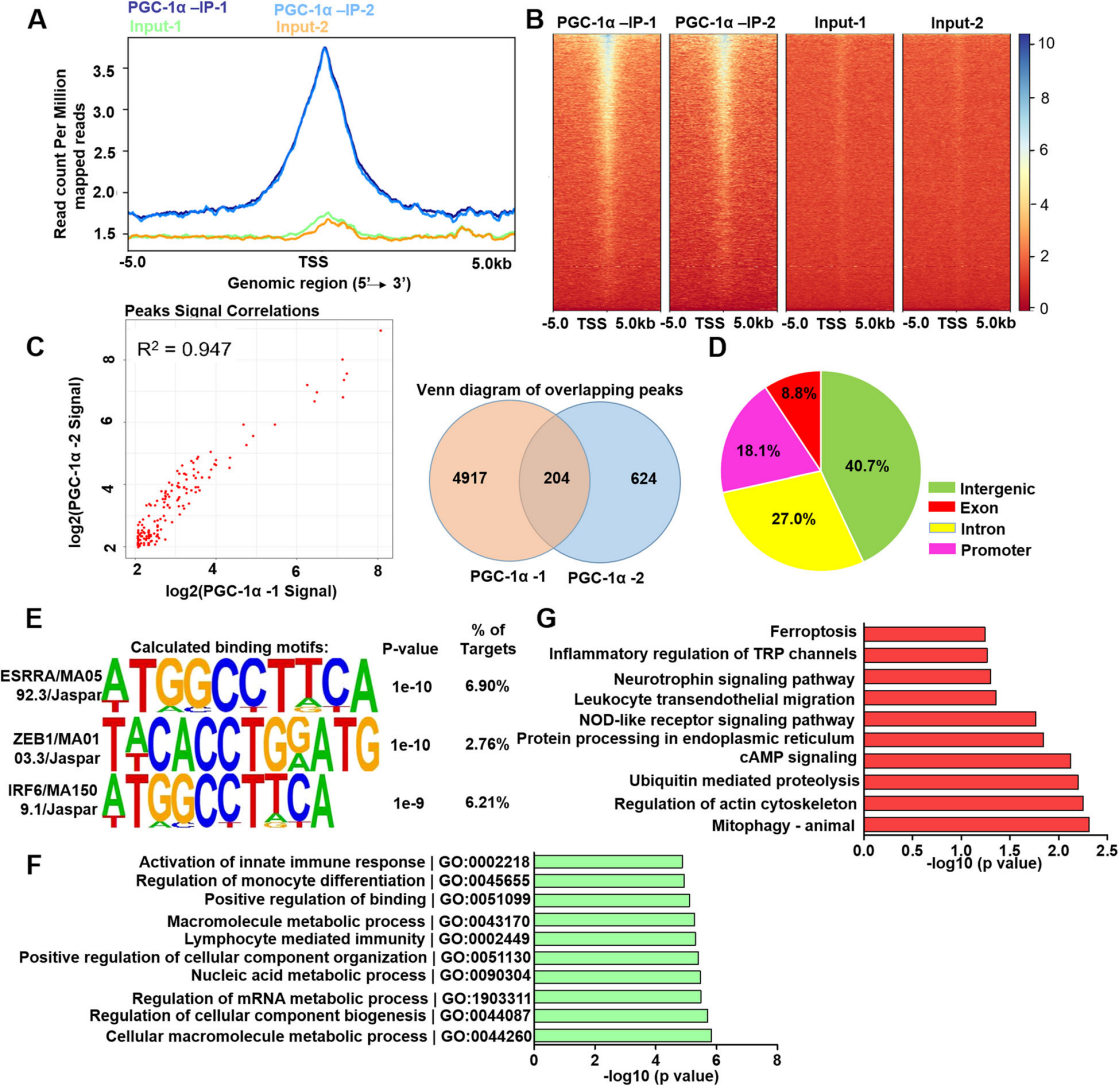
Identification of genome-wide transcriptional targets of PGC-1α. **a** Density distribution of PGC-1α ChIP-Seq peaks. **b** Heat maps of PGC-1α ChIP-Seq and input signals. **c** The intersections between two samples were calculated. Left: A scatterplot visualizing the relationship between the two samples. Right: A Venn diagram displaying the number of overlapping and nonoverlapping peaks between the two samples. **d** Genomic distribution of the transcriptional targets of PGC-1α. **e** De novo motif enrichments identified using HOMER at the overlapping peaks. The top three identified motifs are shown. **f** GO analysis showed the top 10 enriched biological processes. **g** KEGG pathway analysis for the genes that had PGC-1α-binding peaks in their promoter regions (− 2 to 2 kb around TSS)

To further clarify the underlying mechanism of the PGC-1α-mediated modulation of microglial function under ischemic stimuli, we performed ChIP-Seq analysis of two samples. As shown in Fig. 5a, b, the results showed the density distribution of the PGC-1α ChIP-Seq peaks. DeepTools (version 3.4.3) was used for the heatmap plotting. Since there were differences between the aligned base counts from the two samples, the bamCoverage step was normalized in the “RPKM” model. Then, we plotted the heatmap with these normalized data. Next, the overlapping peaks were calculated. After filtering, 204 overlapping peaks were determined and analyzed between the two PGC-1a replicates (Fig. 5c), including 40.7% in intergenic regions, 8.8% in exon regions, 27.0% in intron regions, and 18.1% in promoter regions (Fig. 5d). HOMER motif analysis was conducted to identify the regulatory elements. The known motifs of BORIS, CTCF, and Jun-AP1 were found from our analysis. BORIS, widely studied in tumorigenesis, could function as an immunotherapeutic target to inhibit tumor growth [29]. A previous study also indicated that CTCF deficiency was related to increased inflammatory status [30]. Jun-AP1 was reported to mediate the regulation of the immune response [31]. These studies suggested that PGC-1α might regulate physiological processes by interacting with these transcription factors. The de novo motifs of ERRα, ZEB1, and IRF6 were significantly achieved and selected from the analysis. *P* values for the enrichment score were calculated using a binomial test (Fig. 5e). To further identify whether these motifs bind to PGC-1α directly, we searched these motifs with PGC-1α in the STRING database (https://string-db.org/) by Cytoscape (version 3.7.2). As shown in Additional file 2: Fig. S12, ESRRA/MA0592.3/JASPAR (ERRα) was shown to be directly related to PGC-1α, as well as Ppara, Pparg, Esrrg, and Sirt1, which has been reported previously [32–34]. ZEB1 was shown to be indirectly related to PGC-1α through Sirt1 [35]. ZEB1 was reported to function as a transcription factor associated with microglial regulation in ischemic stroke [15]. As shown, IRF6 might be indirectly related to PGC-1α by acting with Pparg. These findings efficiently determined the interactions and functional binding between PGC-1α and the corresponding targeted genes. GO analysis revealed that most of the enriched biological processes were associated with metabolic processes (Fig. 5f). Most importantly, Kyoto Encyclopedia of Genes and Genomes (KEGG) pathway analysis for these genes showed that the mitophagy signaling pathway was highly enriched (Fig. 5g), supporting the hypothesis that mitophagy might participate in PGC-1α-mediated microglial modulation after ischemic stroke. Altogether, these results showed that microglial PGC-1α probably participates in the immune modulation of stroke by regulating mitophagy.

### PGC-1α promotes autophagic and mitophagic activity in microglia

**Fig. 6:**
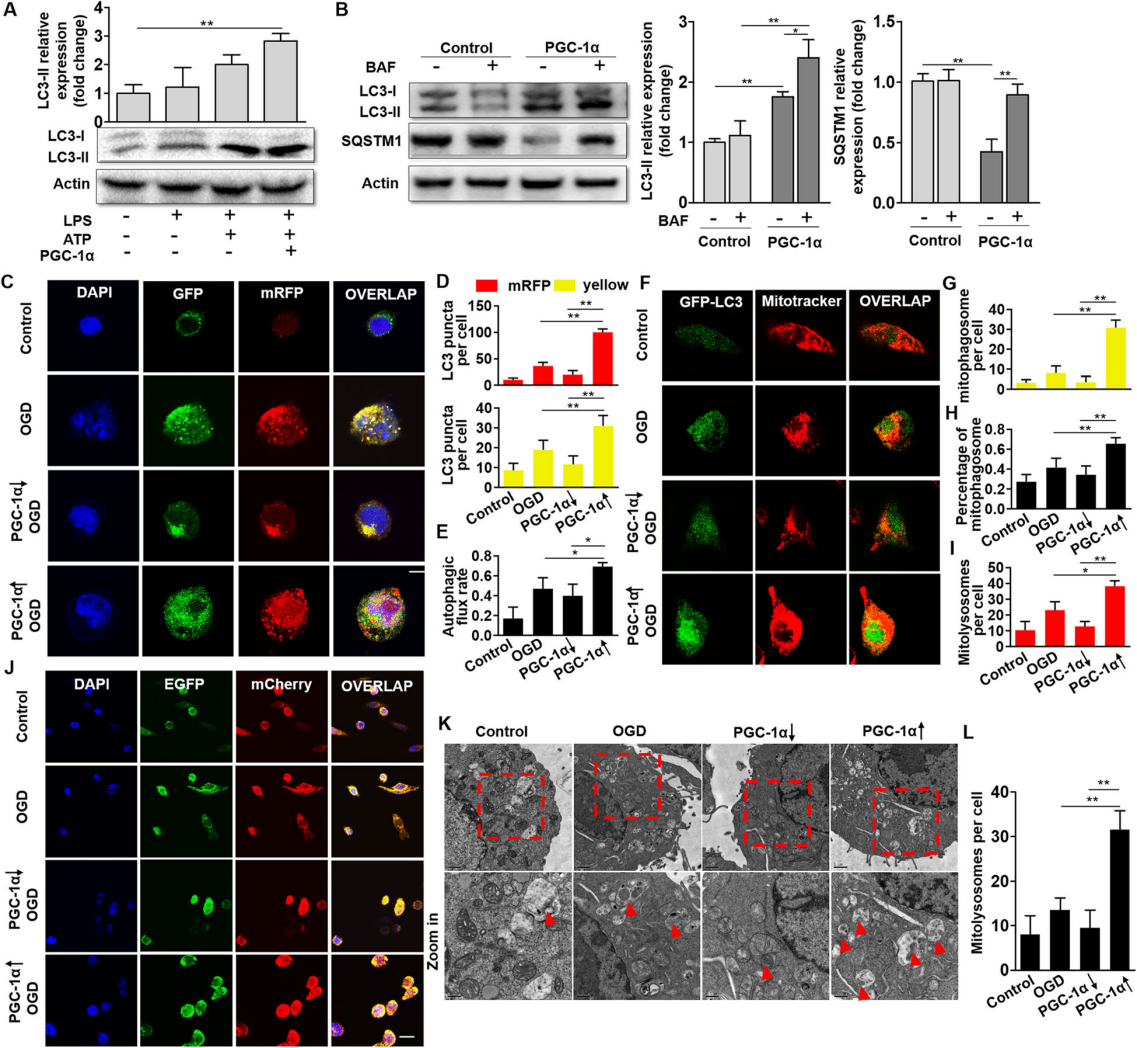
PGC-1α promotes autophagic and mitophagic activity in microglia. **a** Western blot analysis and quantification of the expression of the autophagy-related protein LC3 in LPS-primed BV2 cells after ATP stimulation for 3 h. **b** Assessment of autophagic flux in the presence or absence of the lysosomal inhibitor BAF (50 nM) and determination of LC3-II and SQSTM1 levels. **c** Representative images of mRFP-GFP-LC3 staining showing the autophagosome (yellow) and autolysosome (red-only) formation in the BV2 cells, with or without PGC-1α overexpression after OGD treatment. Bar: 10 μm. **d** Quantification of the number of yellow and red LC3 puncta. **e** Assessment of the autophagic flux rate. **f** Representative images of LC3-MitoTracker colocalization showing mitophagic events in the BV2 cells with or without PGC-1α overexpression after OGD treatment. Bar: 10 μm. **g** Quantification of the number of LC3-MitoTracker colocalized puncta. **h** Quantification of the percentage of LC3-MitoTracker colocalized puncta. **i** Quantification of the number of mitolysosomes. **j** Representative images of the formation of mitolysosomes in the BV2 cells with or without PGC-1α overexpression after OGD treatment. Bar: 20 μm. **k** Representative TEM images of mitolysosomes (red triangle) in the BV2 cells with or without PGC-1α overexpression after OGD treatment. Bar: 1 μm (upper panels), 0.5 μm (lower panels). **l** Quantification of the number of mitolysosomes. *p < 0.05, **p < 0.01; *n* = 6 per group

The autophagic clearance of damaged mitochondria following ischemic stress is important in maintaining mitochondrial homeostasis, thus alleviating neuroinflammation. Autophagic activity under PGC-1α modulation was investigated. The expression of microtubule-associated protein 1 light chain 3 (MAP1LC3), a marker for autophagosome formation, was detected by Western blot and was found to be upregulated in BV2 cells overexpressing PGC-1α (Fig. 6a). Furthermore, the effect of PGC-1α on the autophagic flux of microglia was evaluated. BV2 cells were treated with or without bafilomycin A1 (BAF), a lysosomal inhibitor, to monitor the autophagic flux. We found that the expression of LC3-II was enhanced by PGC-1α induction, and the expression was increased after treatment with BAF (Fig. 6b). Moreover, the expression of SQSTM1, an autophagic biomarker for LC3-II binding, was significantly decreased in the PGC-1α-overexpressing BV2 cells. Additionally, BAF treatment promoted the accumulation of SQSTM1 (Fig. 6b), thus indicating that PGC-1α promoted autophagic flux. Additionally, the dynamic changes in autophagy were further monitored in the BV2 cells transfected with tandem fluorescent-tagged LC3 (Ad-mRFP-GFP-LC3). The dual-color fluorescent probe helped discriminate between autophagosomes (colocalization of GFP with RFP fluorescence, yellow puncta) and autolysosomes (RFP-only signal without GFP, red-only puncta). After OGD treatment, we found that the number of autophagosomes and autolysosomes was increased in the PGC-1α-overexpressing group. The autophagic flux rate was also increased in the PGC-1α-overexpressing cells (Fig. 6c–e). Taken together, these data support the hypothesis that PGC-1α promotes autophagy under ischemic hypoxic stimulus.

To further investigate the essential role of autophagic clearance of damaged mitochondria underlying PGC-1α’s neuroprotective effect, we monitored mitophagic activity. First, activation of mitophagy was observed in BV2 cells overexpressing PGC-1α. BV2 cells were infected with Ad-GFP-LC3 and incubated with MitoTracker Red CMXRos to label autophagosomes and mitochondria. We measured the colocalization of mitochondria (MitoTracker Red) with autophagosomes (GFP) by fluorescence microscopy, indicating the formation of mitophagosomes (MitoTracker^+^/GFP^+^ yellow puncta) (Fig. 6f). The number of mitophagosomes was increased after OGD treatment, and PGC-1α overexpression enhanced the formation of mitophagosomes. However, the downregulation of PGC-1α expression using RNAi dampened the formation of mitophagosomes (Fig. 6g). More specifically, the percentage of mitophagosomes (yellow puncta) in total autophagosomes (GFP puncta) was also increased under PGC-1α overexpression but decreased in the PGC-1α downregulation group (Fig. 6h). These results demonstrated that PGC-1α enhances the initiation of mitophagy in microglia.

Next, we further conducted assays evaluating mitophagic activity at various stages (progression and degradation) under PGC-1α regulation. To visualize the delivery of mitophagosomes to hydrolase-containing lysosomes, we transfected BV2 cells with COX8-EGFP-mCherry lentivirus, a tandem fluorescent-tagged mitochondrial targeting sequence of the inner membrane protein COX8 (cytochrome c oxidase subunit 8). Mitophagosomes appeared as yellow staining of filamentous mitochondria with the merging of both green (EGFP) and red (mCherry) signals, while mitolysosomes appeared to be fragmented with red-only puncta. After OGD treatment, we found that the number of mitolysosomes (mCherry^+^ red puncta) was increased in the PGC-1α-overexpressing group but decreased in the PGC-1α downregulated group (Fig. 6i, j). In addition, TEM was used to further assess the structure of mitochondria, which showed that more mitolysosomes were formed in the PGC-1α-overexpressing BV2 cells, thus indicating that PGC-1α promoted the progression of mitophagy from mitophagosomes to mitolysosomes. However, knockdown of PGC-1α reduced the number of mitolysosomes (Fig. 6k, l). These results indicated that PGC-1α also promotes the progression of mitophagy from mitophagosomes to mitolysosomes.

Mitochondrial degradation and clearance within the lysosome represent the completion of mitophagic flux. The loss of mitochondria was indicated by decreases in multiple mitochondrial proteins, including inner membrane COX4I1 (cytochrome c oxidase subunit 4I1), IMMT/MIC60 (inner membrane mitochondrial protein), and outer membrane TOMM20 (translocase of outer mitochondrial membrane 20). Western blot analysis indicated that lower levels of mitochondrial protein were observed in the PGC-1α-overexpressing group (Additional file 2: Fig. S13). These results suggest that autophagy indeed contributes to the clearance of impaired mitochondria and that PGC-1α increases mitophagic activity and flux in BV2 cells during OGD treatment.

Finally, mitochondrial biogenesis, as well as oxidative metabolic activity, was also evaluated under PGC-1α overexpression. Mitochondrial biogenesis was reflected by oxidative phosphorylation and ATP generation. SOD activity was increased in the mPGC-1α mice compared with the PGC-1α^*f/f*^ mice, while the MDA content was substantially decreased in the mPGC-1α mice after AIS (Additional file 2: Fig. S14a). Under DHE staining, ROS production was found to be significantly decreased in the mPGC-1α mice (Additional file 2: Fig. S14b). Mitochondrial antioxidant and UCP mRNA expression revealed that PGC-1α upregulated the expression of SOD2, Prx3, UCP2, UCP3, UCP4, and UCP5 in the PGC-1α-overexpressing BV2 cells after OGD treatment (Additional file 2: Fig. S14c, d). As expected, PGC-1α also reduced ROS production in BV2 cells (Additional file 2: Fig. S14e). Thus, mitochondrial biogenesis was increased under OGD conditions in the cells overexpressing PGC-1α. To further confirm whether PGC-1α could regulate microglial metabolism, we assessed mitochondrial respiration by measuring the OCR in BV2 cells using a Seahorse assay. After OGD treatment, we also found that basal respiration, ATP production, maximal respiration, and proton leak were significantly increased in the PGC-1α-overexpressing BV2 cells compared to the BV2 cells without PGC-1α overexpression (Additional file 2: Fig. S14f, g). These data indicated that PGC-1α promotes mitophagy and eventually enhances the energetic metabolism of microglia, which in turn generally improves the function of microglia.

### PGC-1α effectively promotes the expression of ULK1 in an ERRα-dependent manner

Genes that were associated with mitophagy from the ChIP-Seq analysis were enriched according to the intensity of the dominant PGC-1α peak of each corresponding gene. Importantly, ULK1 was found to be the most intriguing. Specific information on the peak locations of ULK1 is shown in Additional file 2: Fig. S15a. To validate the ChIP-Seq results, we performed a ChIP-qPCR assay. As expected, ULK1 ChIP achieved a greater than 14-fold enrichment (Additional file 2: Fig. S15b). To further verify the transcriptional regulation of ULK1 under PGC-1α expression, we performed a luciferase assay by transfecting different combinations of plasmids into BV2 cells. As revealed by the above HOMER motif analysis, ERRα was found to be directly related to PGC-1α. Furthermore, ERRα is believed to cooperate with PGC-1α to exert its regulatory effects. Thus, ERRα was introduced to this analysis. Indeed, we found that the relative luciferase activity did not show a significant increase under PGC-1α transfection; however, it was increased in the presence of both PGC-1α and ERRα (Additional file 2: Fig. S15c). Additionally, the activity decreased after treatment with XCT790, an ERRα inverse agonist (Additional file 2: Fig. S15c). Moreover, ULK1 mRNA expression increased in the presence of both PGC-1α and ERRα (Additional file 2: Fig. S15d). In the culture system, we found that PGC-1α could effectively increase ERRα and ULK1 expression in BV2 cells after LPS and ATP stimulation (Additional file 2: Fig. S15e). To evaluate the essential role of ERRα in the beneficial effects of microglial PGC-1α overexpression in cells and mice, we used XCT790, an ERRα inverse agonist, to interfere with the expression of ERRα. IL-1β, TNF-α, and IL-6 were increased in the XCT790-treated BV2 cells under OGD treatment, which suggests that ERRα is necessary for the anti-inflammatory effects of PGC-1α in microglia (Additional file 2: Fig. S15f-h). Next, XCT790 was used to treat the PGC-1α^*f/f*^ and mPGC-1α mice with AIS, and we found that the PGC-1α-induced reduction in neurological deficits could be reversed by XCT790 administration (Additional file 2: Fig. S15i-k). These findings suggest that PGC-1α may regulate ULK1 expression in coordination with ERRα in microglia.

To further assess whether ULK1 participated in PGC-1α-induced mitophagy, we transfected LV-ULK1-RNAi into BV2 cells to inhibit ULK1 expression under PGC-1α overexpression. The formation of mitophagosomes and mitolysosomes was reduced in the LV-ULK1-RNAi-transfected group (Additional file 2: Fig. S16a-c). Additionally, supernatants from cultured BV2 cells were collected for cytokine determination. IL-1β, TNF-α, and IL-6 were increased in the LV-ULK1-RNAi-transfected group (Additional file 2: Fig. S16d-f). The activation of NLRP3 and IL-1β expression were also enhanced when ULK1 was inhibited (Additional file 2: Fig. S16g-i), which abolished the anti-inflammatory effects of PGC-1α. For determination of the effect of ULK1 on microglia, the expression of ULK1 in BV2 cells was controlled by lentiviral transfection. After LPS stimulation, inflammatory cytokines were detected by ELISA. The results showed that proinflammatory cytokines were decreased in the ULK1-overexpressing BV2 cells, while more inflammatory reactions were observed in the ULK1-knockout BV2 cells (Additional file 2: Fig. S17). These data indicated that PGC-1α promotes the expression of ULK1, which is necessary for mediating mitophagic activity and downstream inflammatory responses.

### Pharmacological inhibition or knockdown of ULK1 abolishes the neuroprotective effects of PGC-1α in the mPGC-1α mice

To evaluate the effects of autophagy and mitophagy in vivo, we performed intervention experiments in the tMCAO model. More specifically, hydroxychloroquine (CQ), an autophagic inhibitor, was used to treat the mice with ischemic stroke for three consecutive days (Additional file 2: Fig. S18a). We found that the PGC-1α-induced attenuation of neurological deficits could be reversed by CQ administration (Additional file 2: Fig. S18b). However, there was no obvious aggravation in neurological deficits in the PGC-1α^*f/f*^ mice treated with an autophagic inhibitor, which suggests that the increased autophagy may mediate the effects of PGC-1α in microglia. In addition, lentivirus carrying ULK1 RNAi was injected into the cortex of the transgenic mice to reduce ULK1 expression at day 7 before tMCAO surgery (Additional file 2: Fig. S18c). We found that reducing ULK1 reversed the neuroprotective effects of PGC-1α on the mPGC-1α mice (Additional file 2: Fig. S18d). The mitochondrial proteins (as indicated by COX4I1, IMMT/MIC60, and outer membrane TOMM20) of microglia isolated from mPGC-1α and ULK1 lentivirus-injected mice were evaluated by Western blots, which indicated that autophagic clearance of mitochondria was impaired under ULK1 downregulation (Additional file 2: Fig. S18e, f). Altogether, these results suggest that the neuroprotective effects of microglial PGC-1α may be mediated by autophagy and mitophagy through ULK1 after ischemic stroke.

## Discussion

In this study, we discovered a previously unknown role of microglial PGC-1α in AIS. Our data showed that PGC-1α attenuated ischemia-induced neuroinflammation and neural damage. Moreover, these effects were mainly mediated by PGC-1α-induced autophagy and mitophagy through regulation of ULK1 in an ERRα-dependent manner. This process, in turn, leads to the inhibition of NLRP3 inflammasome-mediated inflammatory responses (Additional file 2: Fig. S19). Thus, our findings highlight an important neuroprotective role of PGC-1α in microglia after AIS.

The protective effects of PGC-1α on neurons [36] and astrocytes [14] have been well studied. A previous study suggested that a PGC-1-related coactivator could regulate microglial polarization by cooperating with STAT6 [37]. In addition, resveratrol, a polyphenol compound that has antioxidant properties, promotes microglial polarization towards an anti-inflammatory phenotype and inhibits LPS-induced neuroinflammation. Importantly, resveratrol achieves this mainly by increasing PGC-1α expression [38]. Moreover, the balasubramide derivative 3C was shown to suppress microglial activation by enhancing the PGC-1α level in microglia [39]. However, the precise role of PGC-1α in microglia has not been completely elucidated, especially in relation to brain ischemia. To this end, we specifically engineered PGC-1α expression in microglia by generating a transgenic mouse strain. We found that PGC-1α overexpression in microglia attenuated neurological dysfunction after tMCAO. A previous study indicated that microglia could constantly sense the surrounding microenvironment by repeatedly extending and retracting their branches [40]. However, inhibition of THIK-1 decreases microglial ramifications, leading to worse neurological outcomes after stroke [4]. In contrast, ramified microglia could promote neuronal survival after hippocampal excitotoxicity [41]. Similarly, the microglia in CX3CR1-deficient mice have more ramified morphologies, tend to develop anti-inflammatory phenotype polarization, and cause smaller brain infarcts [42]. A previous study also indicated that a more ramified morphology of microglia with upregulated ZEB1 expression may enable microglia to react more accurately and quickly to a stimulus, resulting in improved regulation of inflammatory responses after ischemic stroke [15]. Importantly, ramified microglia could also acquire a less reactive phenotype and regulate the microenvironment in the brain. The improved brain environment reduced neuronal damage after ischemic stroke, thereby contributing to the neuroprotective effects. In our study, we observed that the microglia in the cortex of the mPGC-1α mice appeared to have more ramifications, and PGC-1α might allow microglia to more quickly respond to ischemic damage by enhancing these ramified processes, thereby improving neurological functions after stroke. Our microarray analysis confirmed that PGC-1α altered the gene expression profiles of microglia, which are closely related to immune system processes and anatomical structure formation involved in morphogenesis. These results strongly indicate that PGC-1α mediates the immune modulation of microglia and extensively participates in inflammatory responses under ischemic stroke.

Neuroinflammation is a major pathological process after ischemic stroke and significantly promotes the development of brain damage [43]. We observed that IL-1β dramatically decreased in microglia from the mPGC-1α mice after tMCAO. The maturation and release of IL-1β are mainly regulated by the NLRP3 inflammasome [28, 44], which plays an important role in many inflammatory diseases, such as atherosclerosis and autoimmune diseases [45, 46]. Additionally, growing evidence has shown that NLRP3 produced by microglia is responsible for promoting neuroinflammation [47, 48] and can be suppressed by the activation of mitophagy [49–51]. The suppression of NLRP3 in PGC-1α-overexpressing microglia was observed in our study. In addition to IL-1β, CCL5 expression was decreased in the PGC-1α-overexpressing microglia. CCL5 was shown to decrease the production of IL-10 and contributes to the microglial proinflammatory profile [52]. We speculated that PGC-1α inhibited the activation of microglia, thereby reducing the production of CCL5. The transcription and expression of CCL5 might be indirectly affected by PGC-1α modulation in microglia.

To uncover the precise mechanism of PGC-1α regulation in microglia, we performed ChIP-Seq analysis to identify the genome-wide PGC-1α-binding sites in microglia, and further KEGG pathway analysis revealed that mitophagy was the most significantly enriched signaling pathway. Previous studies have indicated that PGC-1α is involved in regulating mitophagy [10, 53]; however, its role in modulating microglial function was newly discovered in our study. Further functional analysis confirmed that ULK1 was the direct target gene for PGC-1α, which was consistent with the reported role of ULK1 in regulating mitophagy [54–56]. Next, the immunomodulatory mechanism of PGC-1α-ULK1-initiated autophagy and mitophagy was further elucidated. The formation of autophagosomes and autolysosomes was induced by PGC-1α overexpression and reduced after knockdown of ULK1, indicating that ULK1 participates in regulating PGC-1α-induced autophagic flux. In addition, we found that PGC-1α enhanced the formation of mitolysosomes mediated by ULK1. The effect of mitophagy-mediated downstream signal transduction has been well established in other immune cells, and this process eventually leads to reduced inflammation [49, 51].

## Conclusions

In conclusion, our results provide evidence that microglial PGC-1α plays a pivotal role in protecting against ischemia-induced brain injury through the regulation of autophagy and mitophagy and the suppression of neuroinflammation. The role we uncovered for PGC-1α in pathological events after ischemic stroke might identify new therapeutic strategies involving manipulation of PGC-1α in CNS inflammation.

## Supplementary Information

**Additional file 1: Supplementary Tables. Table S1.** Primers used in ChIP-qPCR analysis. Table S2. Primers used in qRT-PCR analysis.

**Additional file 2: Supplementary Figures. Figure S1.** PGC-1α decreases in neurons and astrocytes after AIS. **Figure S2.** Generation of PGC-1α^*f/f*^ Cx3cr1-Cre/ER (mPGC-1α) transgenic mice. **Figure S3.** PGC-1α is specifically overexpressed in microglia. Figure S4. rCBF is not significantly different between the PGC-1α^*f/f*^ and mPGC-1α mice. **Figure S5.** Knockout of PGC-1α exacerbates ischemic brain injury. **Figure S6.** Microglial PGC-1α affects the long-term neurologic functional recovery in mice with AIS. **Figure S7.** Microglia with PGC-1α overexpression protect neurons against inflammatory insults. **Figure S8.** PGC-1α regulates morphology of microglia in mice with AIS. **Figure S9.** PGC-1α overexpression alters the gene profiles of microglia after AIS. **Figure S10.** PGC-1α overexpression alters the gene profiles of BV2 cells after LPS stimulation. (A) Cluster analysis for the differentially expressed mRNAs of BV2 cells with or without PGC-1α overexpression after LPS stimulation. (B) GO analysis showed the top 5 enriched biological processes. (C) KEGG pathway analysis for the top 5 pathways. *n* = 3 per group. **Figure S11.** PGC-1α exerts the anti-inflammatory effects in microglia only under inflammatory insults. **Figure S12.** Validation of the interaction of PGC-1α with the corresponding transcription factors from de novo motif analysis. **Figure S13.** PGC-1α promotes the clearance of damaged mitochondria in microglia. **Figure S14.** PGC-1α regulates mitochondrial biogenesis and suppresses ROS production. **Figure S15.** PGC-1α promotes the expression of ULK1 in an ERRα-dependent manner. **Figure S16.** ULK1 is responsible for the PGC-1α-induced mitophagy. **Figure S17.** ULK1 is responsible for PGC-1α-mediated suppression of inflammation. **Figure S18.** Pharmacological inhibition or knockdown of ULK1 reverses the neuroprotective effect of PGC-1α after ischemic stroke. **Figure S19.** Microglial PGC-1α protects against ischemic brain injury by suppressing neuroinflammation.

## Data Availability

The transcriptome data for microglia and BV2 cells generated during this current study were deposited to the GEO database, accession GSE124874 https://www.ncbi.nlm.nih.gov/geo/query/acc.cgi?acc=GSE124874 [18], GSE152871 https://www.ncbi.nlm.nih.gov/geo/query/acc.cgi?acc=GSE152871 [19], and GSE152769 https://www.ncbi.nlm.nih.gov/geo/query/acc.cgi?acc=GSE152769 [20]. The ChIP-Seq data for microglia were deposited to the GEO database, accession GSE165564 https://www.ncbi.nlm.nih.gov/geo/query/acc.cgi?acc=GSE165564 [25].

## Abbreviations

AIS: Acute ischemic stroke
PGC-1α: PPARγ coactivator-1α
tMCAO: Transient middle cerebral artery occlusion
CNS: Central nervous system
ERRα: Estrogen-related receptor α
ROS: Reactive oxygen species
UCPs: Uncoupling proteins
PD: Parkinson’s disease
ULK1: Unc-51 like kinase 1
eGFP: Enhanced GFP
qRT-PCR: Quantitative real-time polymerase chain reaction
ChIP-Seq: Chromatin immunoprecipitation-sequencing
OGD: Oxygen and glucose deprivation
TEM: Transmission electron microscopy
ELISA: Enzyme-linked immunosorbent assay
TAM: Tamoxifen
FACS: Fluorescence-activated cell sorting
TTC: 2,3,5-Tripenyltetrazolium chloride
GO: Gene Ontology
KEGG: Kyoto Encyclopedia of Genes and Genomes
MAP1LC3: Microtubule-associated protein 1 light chain 3
BAF: Bafilomycin A1
SQSTM1: Sequestosome 1
COX4I1: Cytochrome c oxidase subunit 4I1
IMMT/MIC60: Inner membrane mitochondrial protein
TOMM20: Translocase of outer mitochondrial membrane 20
CQ: Hydroxychloroquine
GEO: Gene Expression Omnibus
rCBF: Regional cerebral blood flow
DCFH-DA: Dichlorodihydrofluorescein-diacetate
DHE: Dihydroethidium
OCR: Oxygen consumption rate
