# Supplementary material for: Microglial PGC-1α protects against ischemic brain injury by suppressing neuroinflammation
Source: Genome Med. 2021 Mar 26;13:47. doi: 10.1186/s13073-021-00863-5 (PMC8004413; doi:10.1186/s13073-021-00863-5)
Supplement: Supplementary file 1 — Additional file 1: Supplementary Tables. Table S1. Primers used in ChIP-qPCR analysis. Table S2. Primers used in qRT-PCR analysis. [file 13073_2021_863_MOESM1_ESM.pdf]

Supplementary Tables

Microglial PGC-1α protects against ischemic brain injury by suppressing neuroinflammation

Bin Han[1,2#], Wei Jiang[2#], Pan Cui[2#], Kai Zheng[2], Chun Dang[2], Junjie Wang[2], He Li[2], Lin Chen[2], Rongxin Zhang[3], Qing Mei Wang[4], Zhenyu Ju[5] and Junwei Hao[1,2]*

[1]Department of Neurology, Xuanwu Hospital, Capital Medical University, Beijing, 100053, China

[2]Department of Neurology, Tianjin Neurological Institute, Tianjin Medical University General Hospital, Tianjin, 300052, China

[3]Laboratory of Immunology and Inflammation, Department of Immunology and Research Center of Basic Medical Sciences, Key Laboratory of Immune Microenvironments and Diseases of Educational Ministry, Tianjin Medical University, Tianjin 300070, China

[4]Stroke Biological Recovery Laboratory, Department of Physical Medicine and Rehabilitation, Spaulding Rehabilitation Hospital, the teaching affiliate of Harvard Medical School Charlestown, MA, 02129, USA

[5]Key Laboratory of Regenerative Medicine of Ministry of Education, Institute of Aging and Regenerative Medicine, Jinan University, Guangzhou, 510632, China

[#] These authors contributed equally to this study.

*Correspondence: haojunwei@vip.163.com

Table S1. Primers used in ChIP-qPCR analysis

Gene	Forward	Reverse
ULK1	CCAGGATGGAGTCCCAGGTTAG	CGCCAGCCAGCACAGGATT

Table S2. Primers used in qRT-PCR analysis

Gene	Forward	Reverse
PGC-1α	ATGAATGCAGCGGTCTTAGC	GGTCATCGTTTGTGGTCAGA
SOD2	CAGACCTGCCTTACGACTATGG	CTCGGTGGCGTTGAGATTGTT
Prx3	GGTTGCTCGTCATGCAAGTG	CCACAGTATGTCTGTCAAACAGG
Trx2	TGGGCTTCCCTCACCTCTAAG	CCTGGACGTTAAAGGTCGTCA
GPx1	GTCTCTCTGAGGCACGATCCG	TTCCGCAGGAAGGTAAACAGC
UCP2	CAGGTCACTGTGCCCTTACCAT	CACTACGTTCCAGGATCCCAAG
UCP3	GGATTTGTGCCCTCCTTTCTG	AGATTCCCGCAGTACCTGGACT
UCP4	GAATGCCTATCGCCGAGGA	AGTAGGAACTTGCTCGTCCGG
UCP5	TCCCAACTGCTCAGCGTG	GGTGCTTCTTGGTAATATCATAAACG
IL-1β	GCTGAAAGCTCTCCACCTCA	AGGCCACAGGTATTTTGTCG
IL-6	CCGGAGAGGAGACTTCACAG	TCTGCAAGTGCATCATCGTT
TNF-α	ATGAGAAGTTCCAAATGGCC	TGGTTTGCTACGACGTGGG
iNOS	GAGCTGGGCTGTACAAACCTT	CATTGGAAGTGAAGCGTTTCG
MCP-1	CAGGTCCCTGTCATGCTTCT	TCATTGGGATCATCTTGCTG
IL-4	CGAAGAACACCACAGAGAGTGAGCT	GACTCATTCATGGTGCAGCTTATCG
Arg-1	CTCCAAGCCAAAGTCCTTAGAG	AGGAGCTGTCATTAGGGACATC
TGF-β	TGCGCTTGCAGAGATTAAAA	CGTCAAAAGACAGCCACTCA
IL-10	TGCTATGCTGCCTGCTCTTA	ATGTTGTCCAGCTGGTCCTT
ULK1	TGGAGGTGGCCGTCAAATG	CGCATAGTGTGCAGGTAGTC
GAPDH	TGTGATGGGTGTGAACCACGAGAA	CATGAGCCCTTCCACAATGCCAAA
