# Supplementary material for: Microglial PGC-1α protects against ischemic brain injury by suppressing neuroinflammation
Source: Genome Med. 2021 Mar 26;13:47. doi: 10.1186/s13073-021-00863-5 (PMC8004413; doi:10.1186/s13073-021-00863-5)
Supplement: Supplementary file 2 — Additional file 2: Supplementary Figures. Figure S1. PGC-1α decreases in neurons and astrocytes after AIS. Figure S2. Generation of PGC-1αf/f Cx3cr1-Cre/ER (mPGC-1α) transgenic mice. Figure S3. PGC-1α is specifically overexpressed in microglia. Figure S4. rCBF is not significantly different between the PGC-1αf/f and mPGC-1α mice. Figure S5. Knockout of PGC-1α exacerbates ischemic brain injury. Figure S6. Microglial PGC-1α affects the long-term neurologic functional recovery in mice with AIS. Figure S7. Microglia with PGC-1α overexpression protect neurons against inflammatory insults. Figure S8. PGC-1α regulates morphology of microglia in mice with AIS. Figure S9. PGC-1α overexpression alters the gene profiles of microglia after AIS. Figure S10. PGC-1α overexpression alters the gene profiles of BV2 cells after LPS stimulation. (A) Cluster analysis for the differentially expressed mRNAs of BV2 cells with or without PGC-1α overexpression after LPS stimulation. (B) GO analysis showed the top 5 enriched biological processes. (C) KEGG pathway analysis for the top 5 pathways. n = 3 per group. Figure S11. PGC-1α exerts the anti-inflammatory effects in microglia only under inflammatory insults. Figure S12. Validation of the interaction of PGC-1α with the corresponding transcription factors from de novo motif analysis. Figure S13. PGC-1α promotes the clearance of damaged mitochondria in microglia. Figure S14. PGC-1α regulates mitochondrial biogenesis and suppresses ROS production. Figure S15. PGC-1α promotes the expression of ULK1 in an ERRα-dependent manner. Figure S16. ULK1 is responsible for the PGC-1α-induced mitophagy. Figure S17. ULK1 is responsible for PGC-1α-mediated suppression of inflammation. Figure S18. Pharmacological inhibition or knockdown of ULK1 reverses the neuroprotective effect of PGC-1α after ischemic stroke. Figure S19. Microglial PGC-1α protects against ischemic brain injury by suppressing neuroinflammation. [file 13073_2021_863_MOESM2_ESM.pdf]

Supplementary Figures

Microglial PGC-1α protects against ischemic brain injury by suppressing neuroinflammation

Bin Han[1,2#], Wei Jiang[2#], Pan Cui[2#], Kai Zheng[2], Chun Dang[2], Junjie Wang[2], He Li[2], Lin Chen[2], Rongxin Zhang[3], Qing Mei Wang[4], Zhenyu Ju[5] and Junwei Hao[1,2]*

[1]Department of Neurology, Xuanwu Hospital, Capital Medical University, Beijing, 100053, China

[2]Department of Neurology, Tianjin Neurological Institute, Tianjin Medical University General Hospital, Tianjin, 300052, China

[3]Laboratory of Immunology and Inflammation, Department of Immunology and Research Center of Basic Medical Sciences, Key Laboratory of Immune Microenvironments and Diseases of Educational Ministry, Tianjin Medical University, Tianjin 300070, China

[4]Stroke Biological Recovery Laboratory, Department of Physical Medicine and Rehabilitation, Spaulding Rehabilitation Hospital, the teaching affiliate of Harvard Medical School Charlestown, MA, 02129, USA

[5]Key Laboratory of Regenerative Medicine of Ministry of Education, Institute of Aging and Regenerative Medicine, Jinan University, Guangzhou, 510632, China

[#] These authors contributed equally to this study.

*Correspondence: haojunwei@vip.163.com

Fig. S1

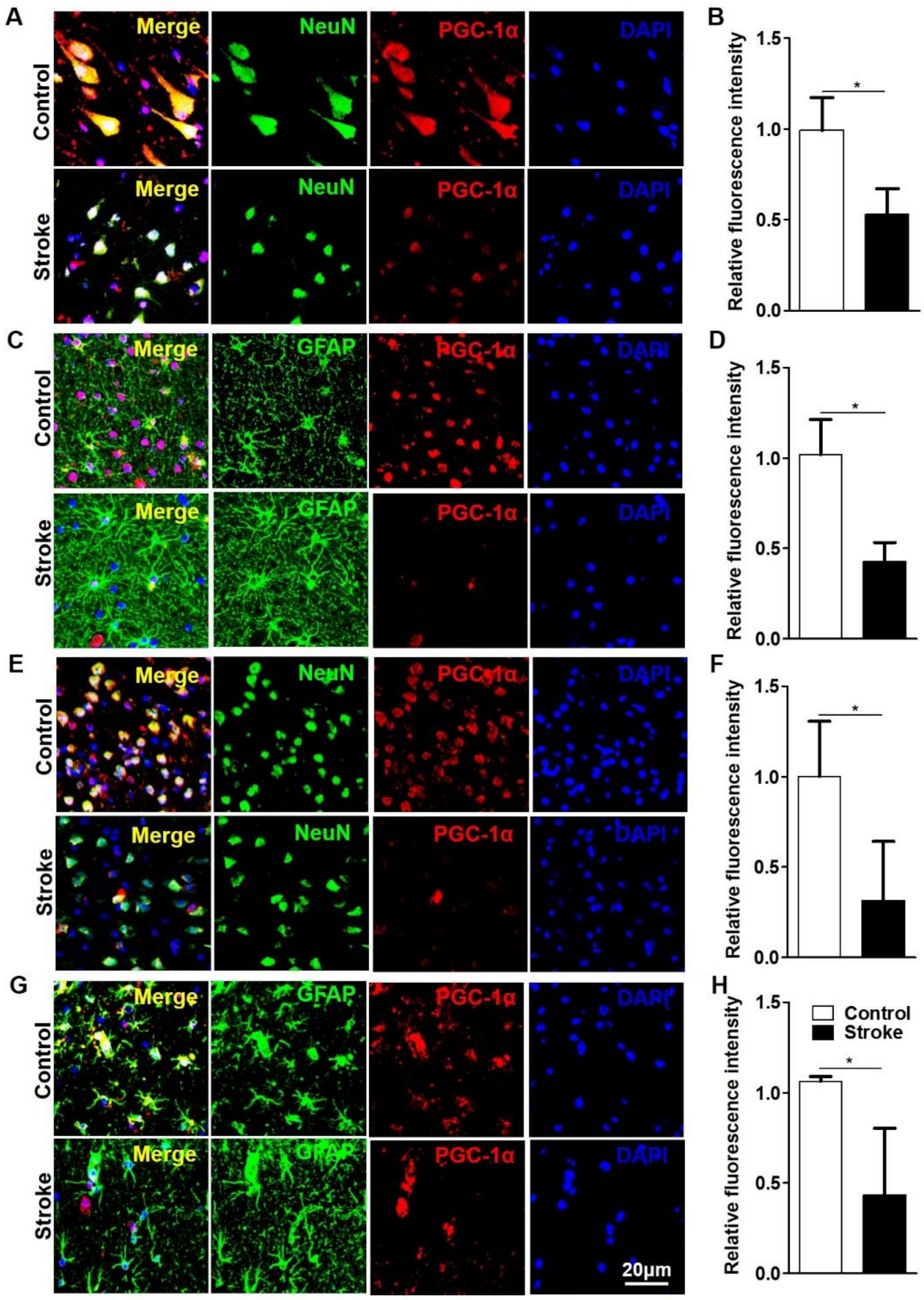

Fig. S1 PGC-1α decreases in neurons and astrocytes after AIS

Representative images (**a**) and quantification of PGC-1α expression (**b**) in neurons from ischemic stroke patients. Representative images (**c**) and quantification of PGC-1α expression (**d**) in astrocytes from ischemic stroke patients. Representative images (**e**) and quantification of PGC-1α expression (**f**) in neurons from ischemic stroke mice. Representative images (**g**) and quantification of PGC-1α expression (**h**) in astrocytes from ischemic stroke mice. *p<0.05; n = 5 per group.

Fig. S2

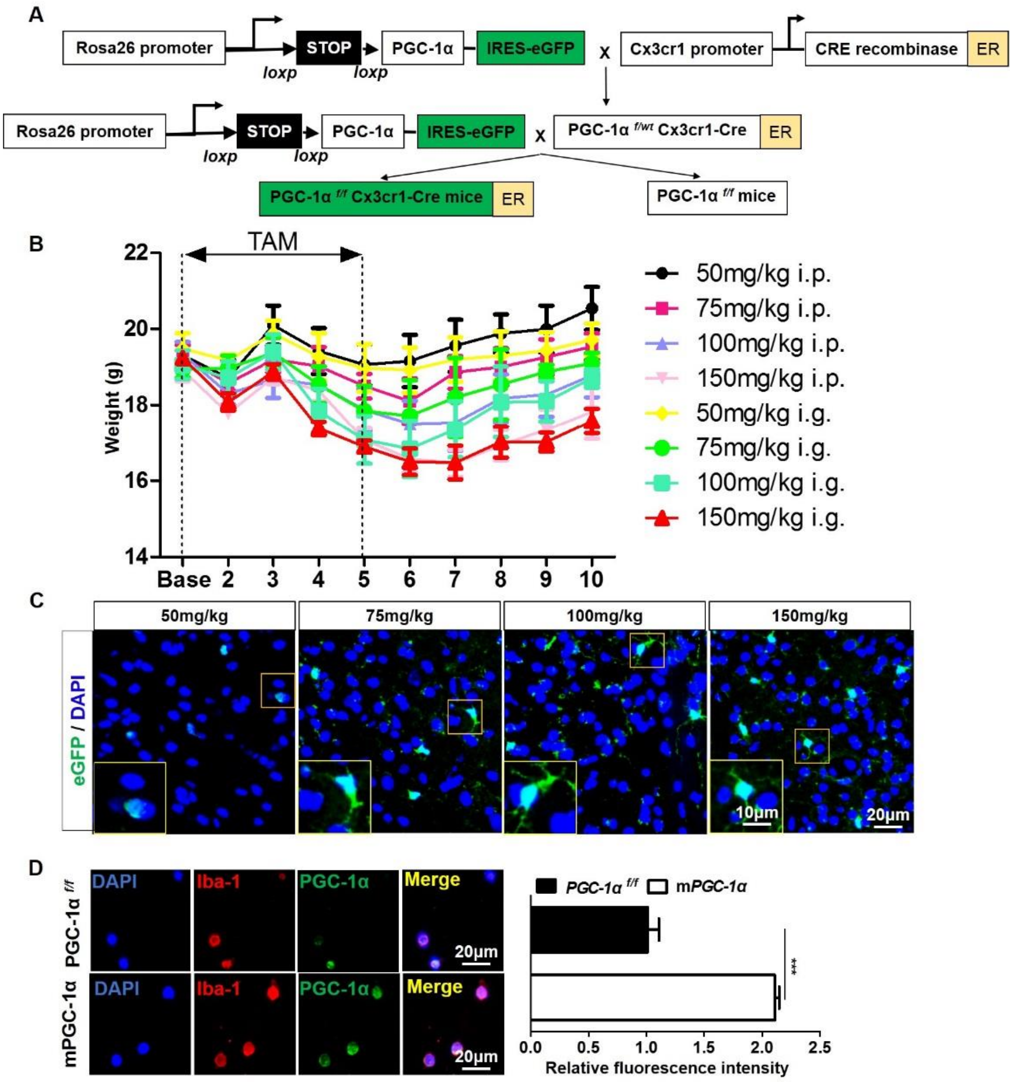

Fig. S2 Generation of PGC-1α$^{f/f}$ Cx3cr1-Cre/ER (mPGC-1α) transgenic mice

a Schematic of the hybrid strategy of generating mPGC-1α transgenic mice. **b** Mean body weight of mice given different doses of tamoxifen either intraperitoneally (i.p.) or gavage (i.g.). **c** Representative images of eGFP immunostaining in the brain sections with TAM administration. **d** Immunostained images showing the PGC-1α expression in microglia from the PGC-1α$^{f/f}$ and mPGC-1α mice after TAM induction (left panel). PGC-1α levels were quantified using ImageJ software (right panel). ***p<0.001, (**b, c**) n = 5 per group, (**d**) n = 8 per group.

Fig. S3

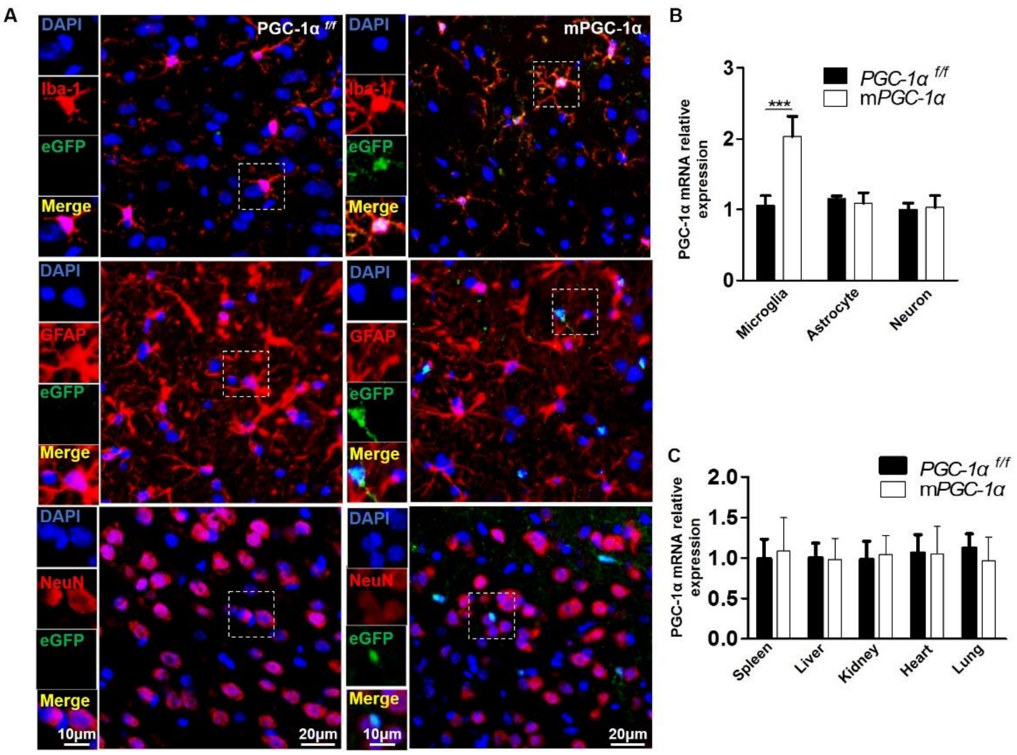

Fig. S3 PGC-1α is specifically overexpressed in microglia

a The location of eGFP was evaluated by costaining with Iba-1, GFAP and NeuN. **b** Quantification of PGC-1α mRNA expression in primary microglia, astrocytes and neurons. **c** Quantification of PGC-1α mRNA expression in the spleen, liver, kidney, heart, and lung. ***p<0.001, n = 5 per group.

Fig. S4

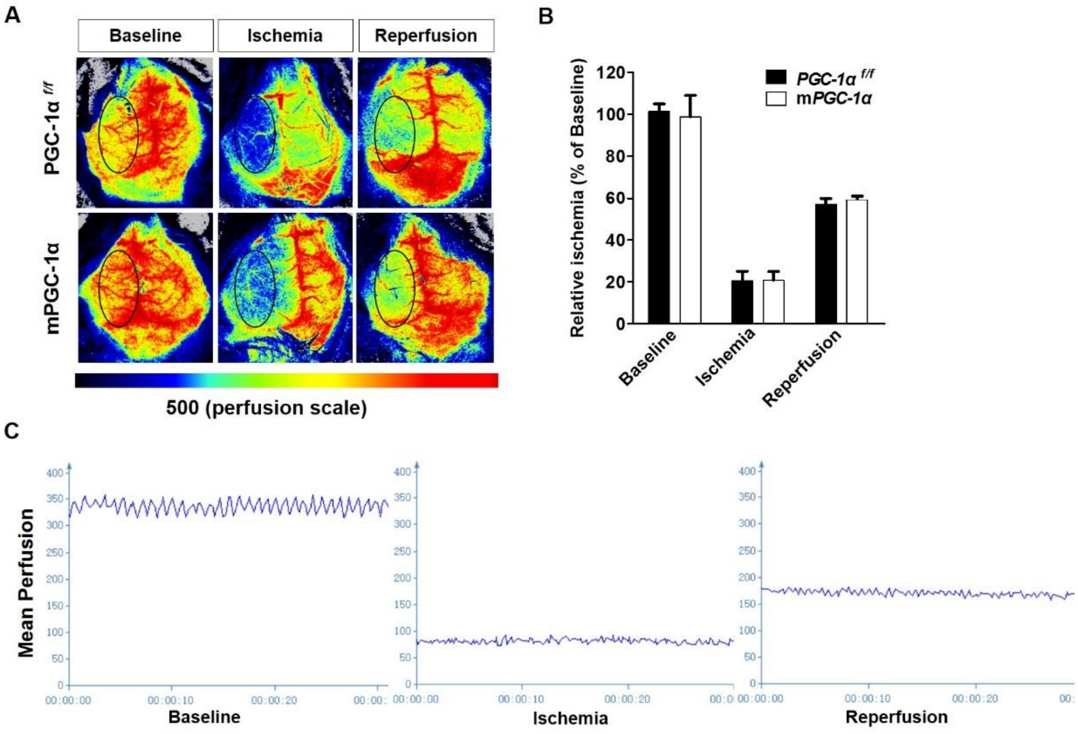

Fig. S4 rCBF is not significantly different between the PGC-1α$^{f/f}$ and mPGC-1α mice

a Representative perfusion images of the PGC-1α$^{f/f}$ and mPGC-1α mice acquired by laser speckle flowmetry at baseline, 30 min after ischemia, and 2 min after reperfusion. **b** Relative rCBF in the two types of mice was presented as the percentage by normalizing baseline. No difference was observed between the two groups. **c** Representative graphs displayed mean perfusion at baseline, ischemia and reperfusion. n = 10 per group.

Fig. S5

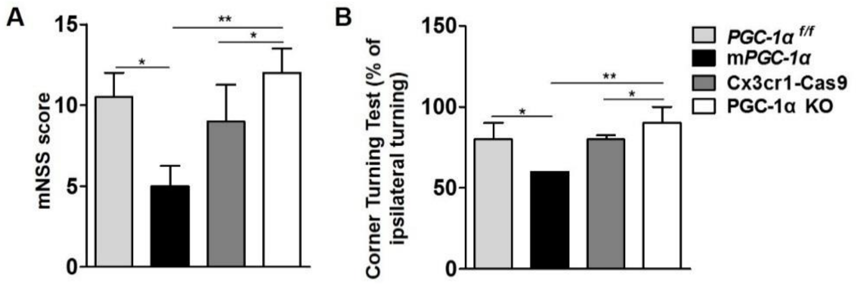

Fig. S5 Knockout of PGC-1α exacerbates ischemic brain injury

a, b Clinical scores of the *PGC-1α*$^{f/f}$, mPGC-1α, Cx3cr1-Cas9 and PGC-1α KO mice at day 3 after tMCAO.

*p<0.05, **p<0.01; n = 6 per group.

Fig. S6

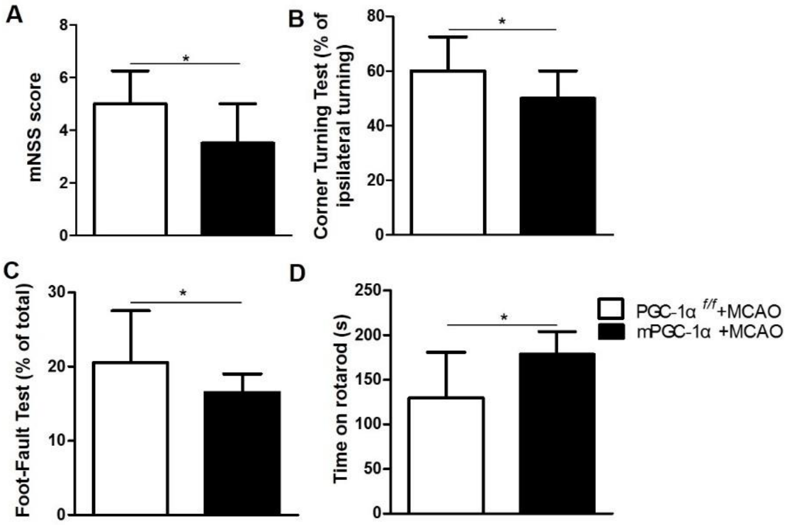

Fig. S6 Microglial PGC-1α affects the long-term neurologic functional recovery in mice with AIS

a-d Analysis of the mNSS score, corner turning test, foot-fault test and time on rotarod in the PGC-1α$^{f/f}$ and mPGC-1α mice with AIS at 2 weeks after tMCAO. *p<0.05; n = 10 per group.

Fig. S7

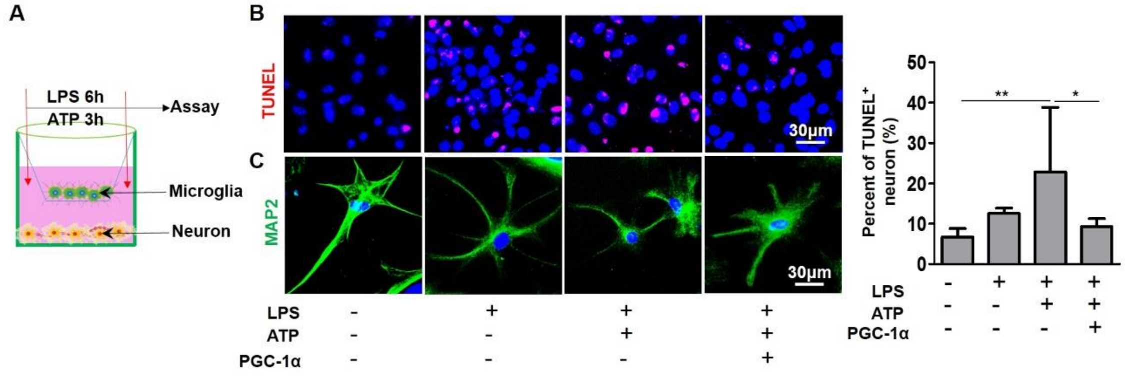

Fig. S7 Microglia with PGC-1α overexpression protect neurons against inflammatory insults

a Schematic diagram of microglia cocultured with neurons. **b** TUNEL staining for neuronal apoptosis and MAP2 staining for neuronal morphology. **c** Quantification of TUNEL-positive neurons (right panel). *p<0.05, **p<0.01; n = 5 per group.

Fig. S8

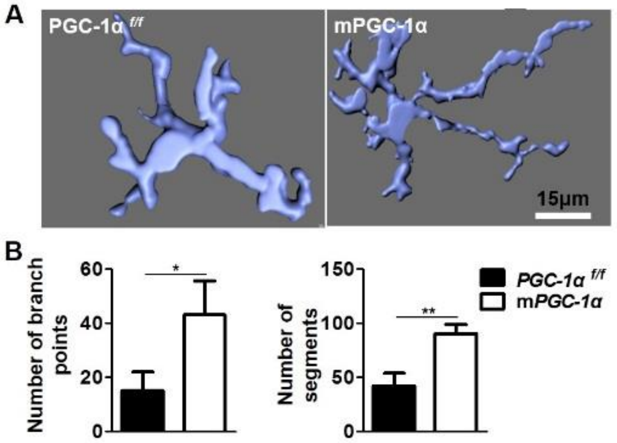

Fig. S8 PGC-1α regulates morphology of microglia in mice with AIS

a Representative images of Imaris-based three-dimensional reconstruction of Iba-1^{+} microglia from the PGC-1α$^{f/f}$ and mPGC-1α mice after AIS. **b** Morphological features including branch points and number of segments were quantified using Imaris. *p<0.05, **p<0.01; n=6 per group.

Fig. S9

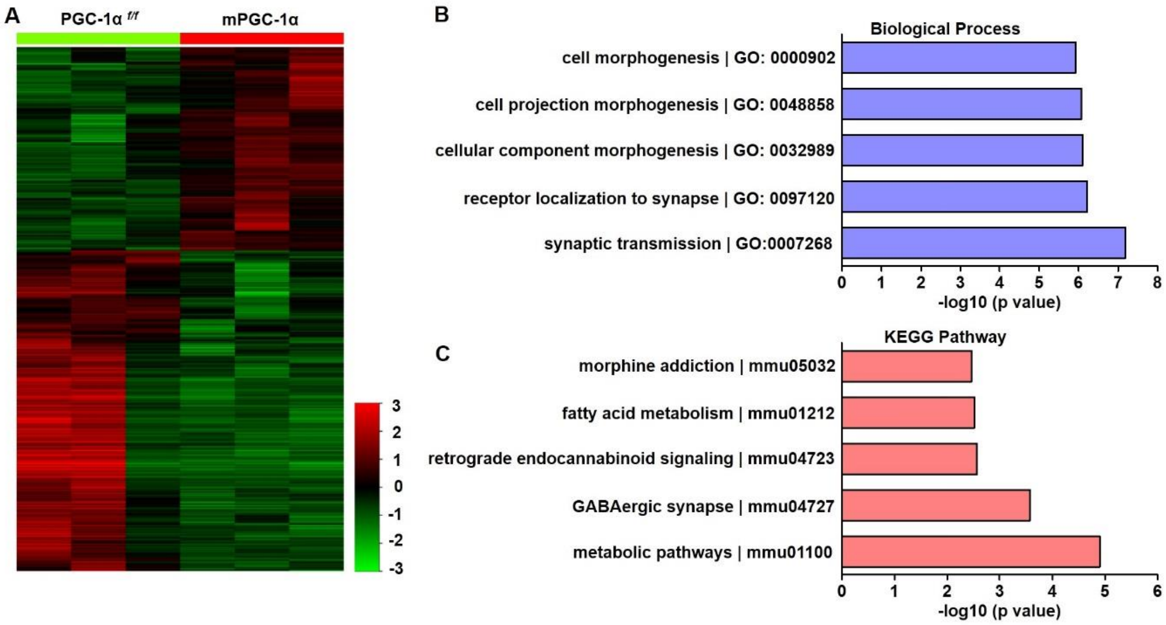

Fig. S9 PGC-1α overexpression alters the gene profiles of microglia after AIS

a Cluster analysis of the differentially expressed mRNAs of microglia from the PGC-1α$^{f/f}$ and mPGC-1α mice after AIS. **b** GO analysis showed top 5 enriched biological processes. **c** KEGG pathway analysis for top 5 pathways. n = 3 per group.

Fig. S10

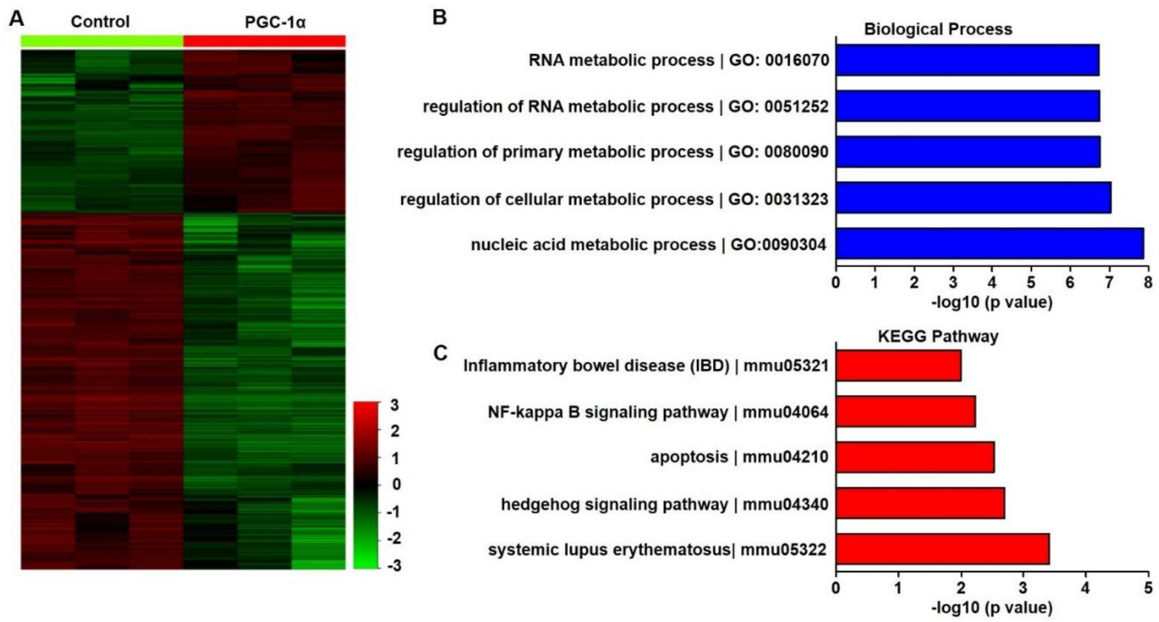

Fig. S10 PGC-1α overexpression alters the gene profiles of BV2 cells after LPS stimulation

a Cluster analysis of the differentially expressed mRNAs of BV2 cells with or without PGC-1α overexpression after LPS stimulation. **b** GO analysis showed top 5 enriched biological processes. **c** KEGG pathway analysis for top 5 pathways. n = 3 per group.

Fig. S11

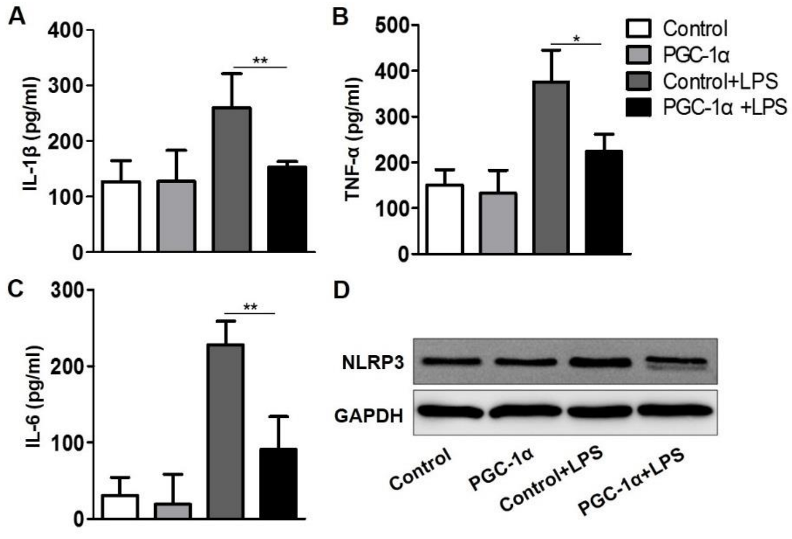

Fig. S11 PGC-1α exerts the anti-inflammatory effects in microglia only under inflammatory insults

ELISA analysis of the levels of IL-1β (**a**), TNF-α (**b**) and IL-6 (**c**) in culture supernatants of BV2 cells with or without LPS stimulation. **d** Western blots showing the expression of NLRP3 in cellular lysates of BV2 cells with or without LPS stimulation. *$p<0.05$, **$p<0.01$; n = 5-10 per group.

Fig. S12

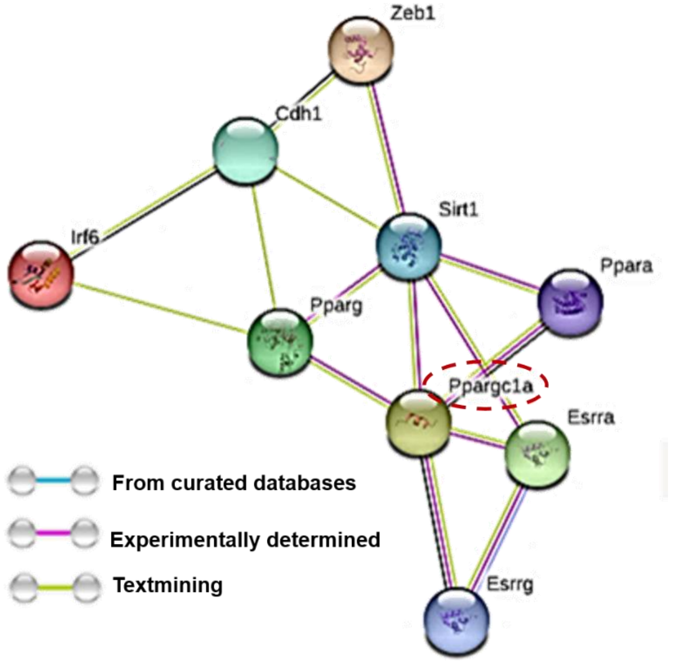

Fig. S12 Validation of the interaction of PGC-1α with the corresponding transcription factors from *de novo* motif analysis

Fig. S13

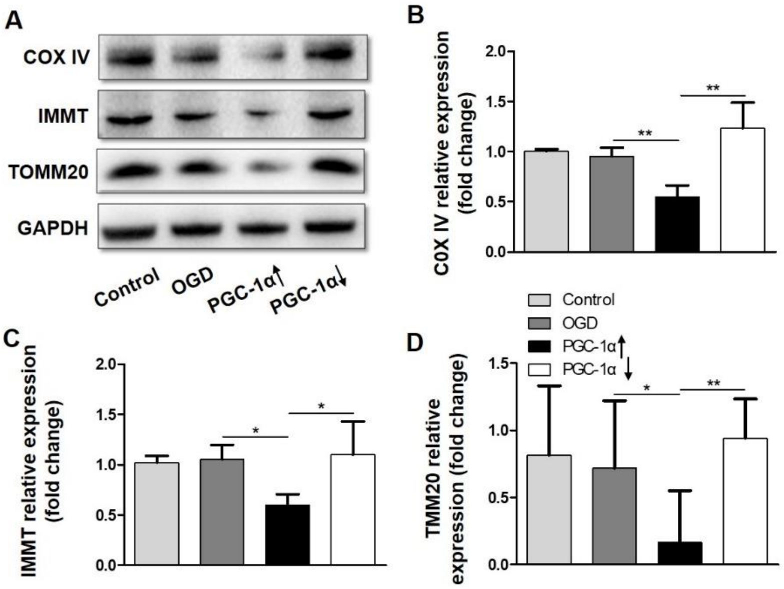

Fig. S13 PGC-1α promotes the clearance of damaged mitochondria in microglia

a Representative images of COX4I1, IMMT/MIC60 and outer membrane TOMM20 bands by Western blot.

(**b-d**) Quantification of the proteins level under PGC-1α modulation. *$p<0.05$, **$p<0.01$; n = 6 per group.

Fig. S14

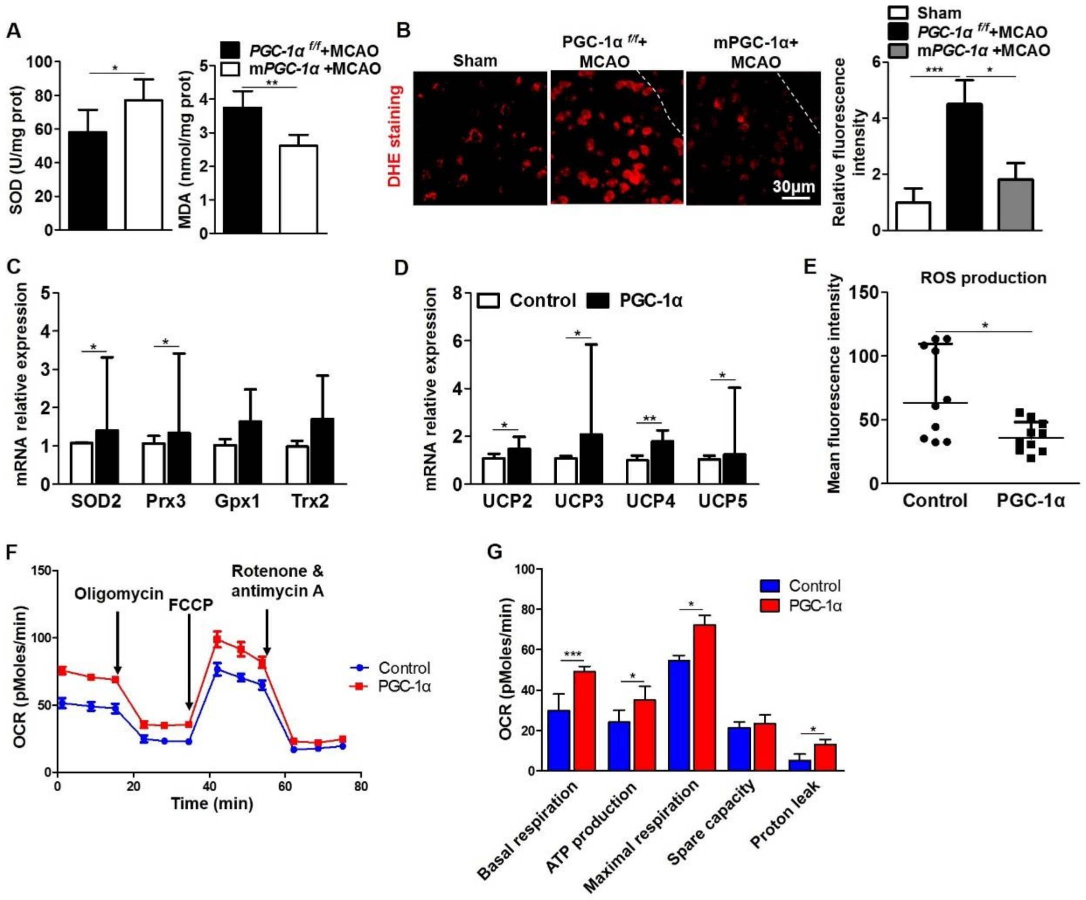

Fig. S14 PGC-1α regulates mitochondrial biogenesis and suppresses ROS production

a Comparisons of SOD activity and MDA levels between the PGC-1α$^{f/f}$ and mPGC-1α mice. **b** Representative images of the ROS level detected by DHE staining for the sham-operated controls, PGC-1α$^{f/f}$ and mPGC-1α mice (left panel). Quantification of relative fluorescence intensity of DHE staining (right panel). The mRNA expression of mitochondrial antioxidants (**c**) and UCPs (**d**) in BV2 cells after OGD stimulation. **e** Quantitation of ROS production in BV2 cells after OGD stimulation. **f** Seahorse assay for the OCR in BV2 cells with or without PGC-1α overexpression. **g** Quantification of basal respiration, ATP production, maximal respiration, spare capacity and proton leak. *p<0.05, **p<0.01, ***p<0.001; n = 7-10 per group.

Fig. S15

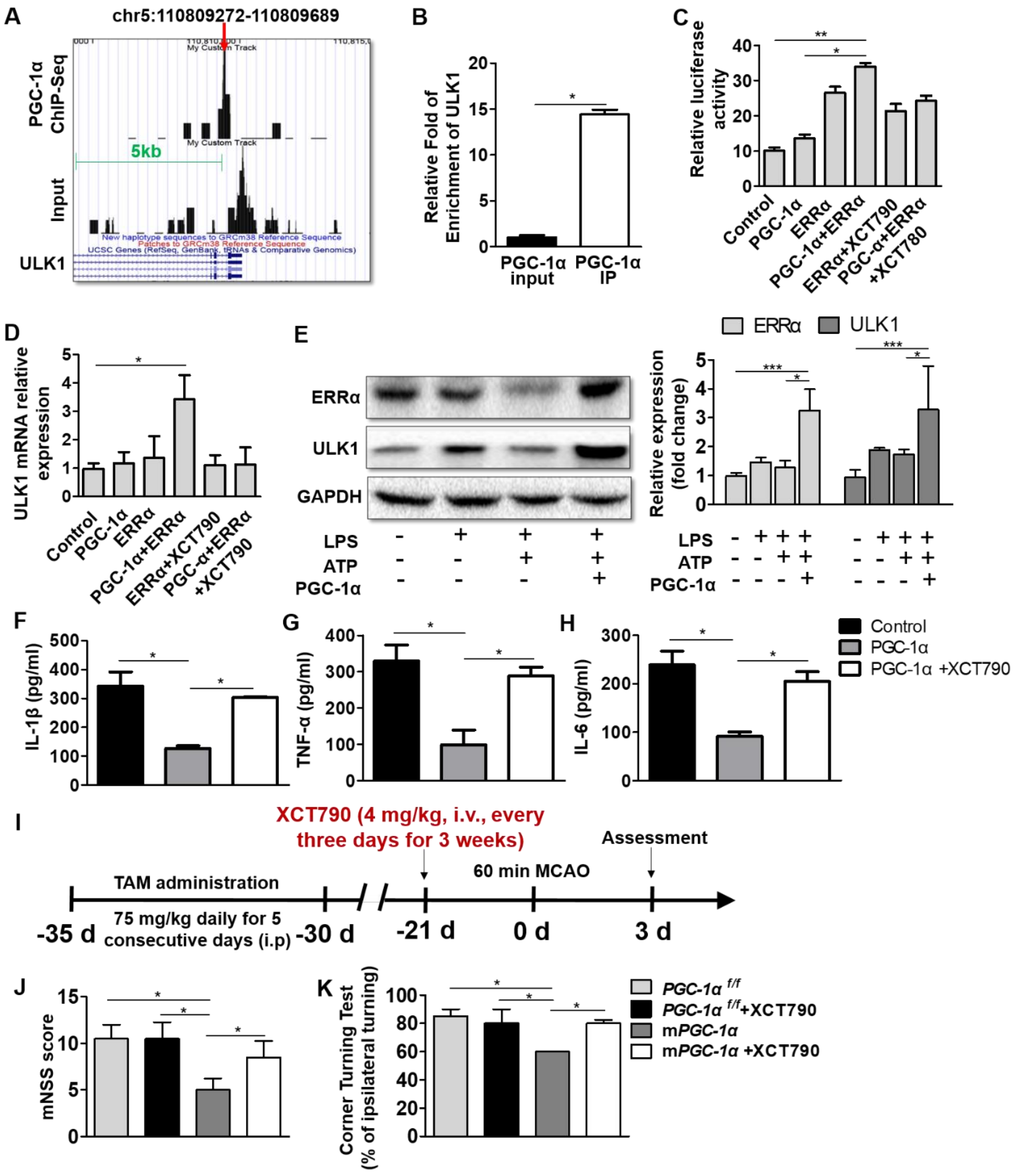

Fig. S15 PGC-1α promotes the expression of ULK1 in an ERRα-dependent manner

a Representative ChIP-Seq peaks of PGC-1α located on *Ulk1* gene in the UCSC Genome browser. **b** ChIP-qPCR analysis for the validations of *Ulk1* enrichment. The relative luciferase activity (**c**) and ULK1 mRNA level (**d**) after transfecting different combinations of plasmids to the BV2 cells with or without

XCT790 (2 μM, 48 h), an ERRα inverse agonist. **e** Western blot analysis and quantification of ERRα and ULK1 expression in BV2 cells with or without PGC-1α overexpression under LPS and ATP treatment. ELISA analysis of the levels of IL-1β (**f**), TNF-α (**g**) and IL-6 (**h**) in culture supernatants of BV2 cells with or without XCT790 after OGD treatment. **i** Schematic of the PGC-1α$^{f/f}$ and mPGC-1α mice treated with an ERRα inverse agonist, XCT790 (4 mg/kg, i.v.), every three days for 3 weeks, followed by neurobehavioral test after tMCAO. **j** Clinical scores of the PGC-1α$^{f/f}$ and mPGC-1α mice with or without CQ treatment. *$p<0.05$, **$p<0.01$, ***$p<0.001$; n = 6 per group.

Fig. S16

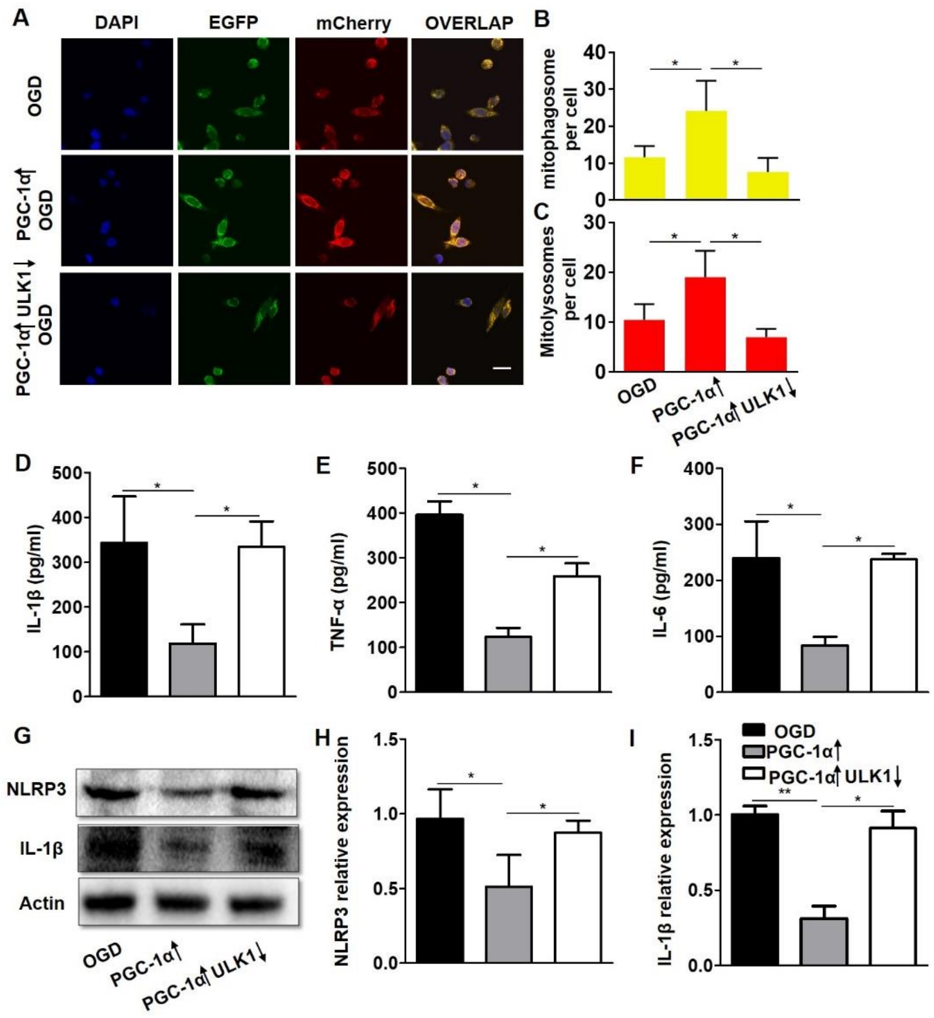

Fig. S16 ULK1 is responsible for the PGC-1α-induced mitophagy

a Representative images of the formation of mitophagosomes and mitolysosomes in the BV2 cells with or without ULK1 downregulation after OGD treatment. Bar: 20 μm. **b** Quantification of the number of mitophagosomes. **c** Quantification of the number of mitolysosomes. ELISA analysis of the levels of IL-1β (**d**), TNF-α (**e**) and IL-6 (**f**) in culture supernatants of BV2 cells with or without ULK1 downregulation after OGD treatment. **g** Western blots showing the expression of NLRP3 and IL-1β in cellular lysates of BV2 cells with or without ULK1 downregulation after OGD treatment. Quantification of the relative expression of NLRP3 (**h**) and IL-1β (**i**). *p<0.05, **p<0.01; n = 6 per group.

Fig. S17

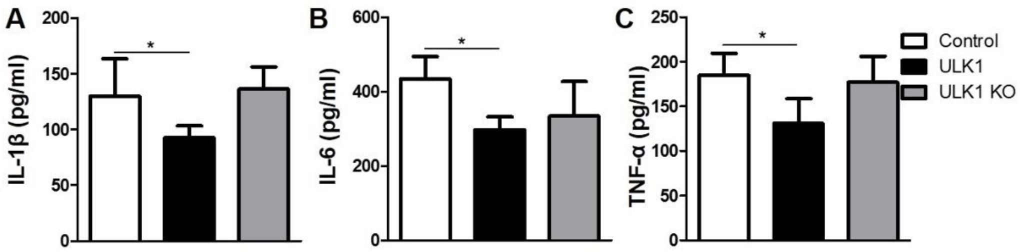

Fig. S17 ULK1 is responsible for PGC-1α-mediated suppression of inflammation

ELISA analysis of the levels of IL-1β (**a**), IL-6 (**b**) and TNF-α (**c**) in culture supernatants of BV2 cells with or without ULK1 overexpression after LPS stimulation. *p<0.05; n = 6 per group.

Fig. S18

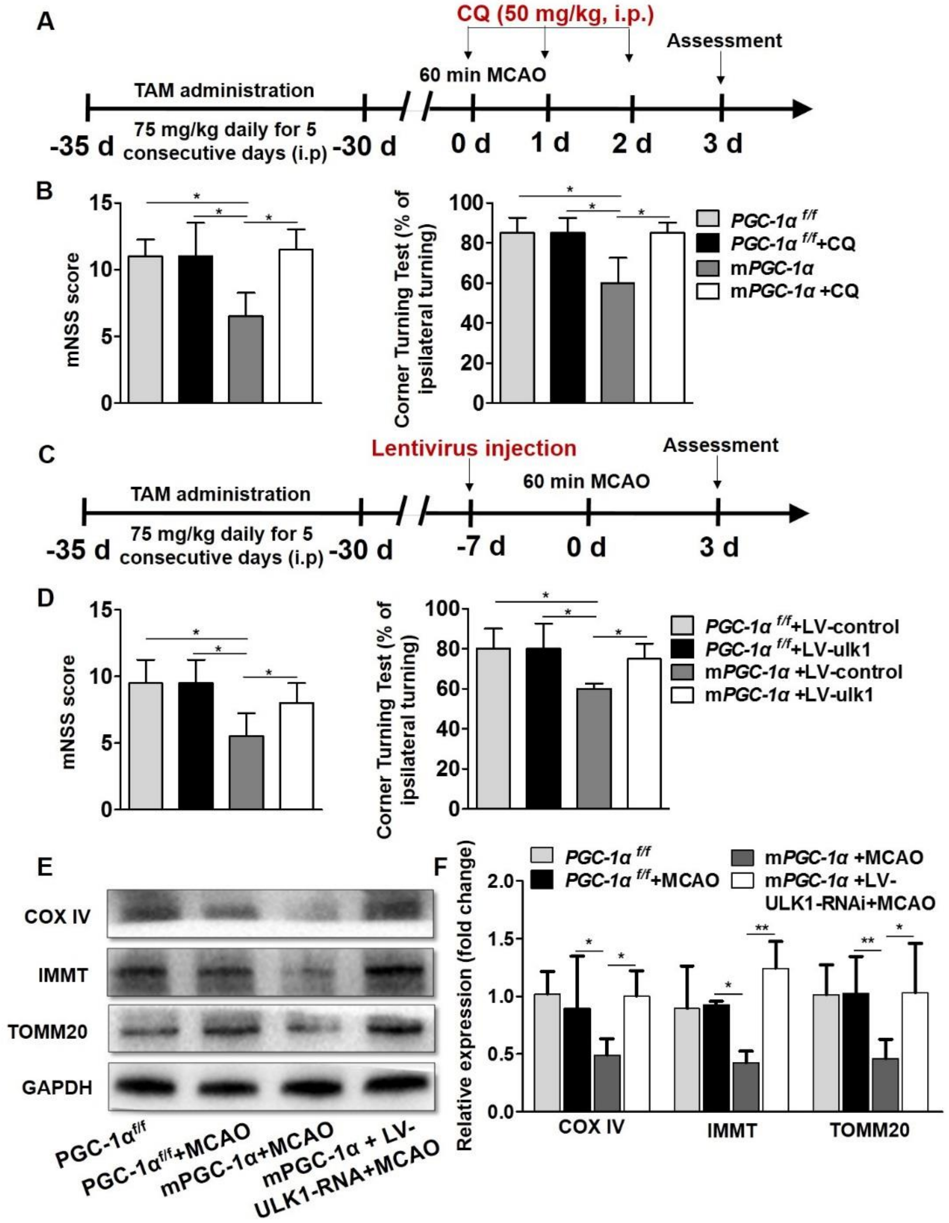

Fig. S18 Pharmacological inhibition or knockdown of ULK1 reverses the neuroprotective effect of PGC-1α after ischemic stroke

a Schematic of the PGC-1α$^{f/f}$ and mPGC-1α mice treated with an autophagy inhibitor CQ (50 mg/kg, i.p.) for 3 consecutive days after tMCAO followed by neurobehavioral test. **b** Clinical scores of the PGC-1α$^{f/f}$ and mPGC-1α mice with or without CQ treatment. **c** Schematic of the PGC-1α$^{f/f}$ and mPGC-1α mice injected with lentiviruses to knockdown ULK1 expression at day 7 before tMCAO followed by neurobehavioral test. **d** Clinical scores of the PGC-1α$^{f/f}$ and mPGC-1α mice treated with or without ULK1 lentiviruses. **e, f** Western blot analysis indicating less mitochondrial proteins in the mPGC-1α mice than PGC-1α$^{f/f}$ control or ULK1 lentivirus injected ones. *$p<0.05$, **$p<0.01$; n = 6 per group.

Fig. S19

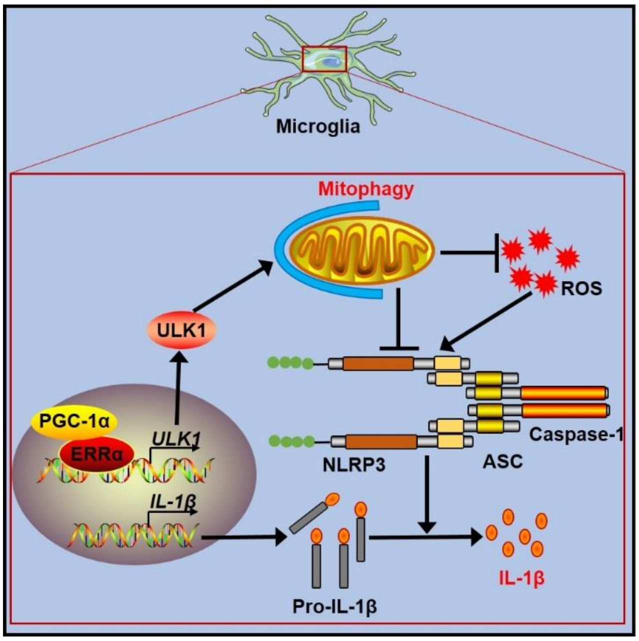

Fig. S19 Microglial PGC-1α protects against ischemic brain injury by suppressing neuroinflammation.

Our data showed that PGC-1α attenuates ischemia-induced neuroinflammation and neural damage. Moreover, these effects were mainly mediated by PGC-1α-induced autophagy and mitophagy through regulation of ULK1 in an ERRα-dependent manner. This, in turn, leads to decreased ROS generation and inhibition of NLRP3-ASC-caspase-1 pathway-mediated inflammatory responses.
